# Human and Murine Toll-like Receptor-Driven Disease in Systemic Lupus Erythematosus

**DOI:** 10.3390/ijms25105351

**Published:** 2024-05-14

**Authors:** Susannah von Hofsten, Kristin Andreassen Fenton, Hege Lynum Pedersen

**Affiliations:** 1 Department of Medical Biology, Faculty of Health Sciences, UiT The Arctic University of Norway, 9019 Tromsø, Norway; susannah.hofsten@uit.no; 2 Centre of Clinical Research and Education, University Hospital of North Norway, Department of Medical Biology, Faculty of Health Sciences, UiT The Arctic University of Norway, 9019 Tromsø, Norway; kristin.fenton@uit.no

**Keywords:** systemic lupus erythematosus, toll-like receptor, mouse models

## Abstract

The pathogenesis of systemic lupus erythematosus (SLE) is linked to the differential roles of toll-like receptors (TLRs), particularly TLR7, TLR8, and TLR9. TLR7 overexpression or gene duplication, as seen with the Y-linked autoimmune accelerator (*Yaa*) locus or TLR7 agonist imiquimod, correlates with increased SLE severity, and specific TLR7 polymorphisms and gain-of-function variants are associated with enhanced SLE susceptibility and severity. In addition, the X-chromosome location of *TLR7* and its escape from X-chromosome inactivation provide a genetic basis for female predominance in SLE. The absence of TLR8 and TLR9 have been shown to exacerbate the detrimental effects of TLR7, leading to upregulated TLR7 activity and increased disease severity in mouse models of SLE. The regulatory functions of TLR8 and TLR9 have been proposed to involve competition for the endosomal trafficking chaperone UNC93B1. However, recent evidence implies more direct, regulatory functions of TLR9 on TLR7 activity. The association between age-associated B cells (ABCs) and autoantibody production positions these cells as potential targets for treatment in SLE, but the lack of specific markers necessitates further research for precise therapeutic intervention. Therapeutically, targeting TLRs is a promising strategy for SLE treatment, with drugs like hydroxychloroquine already in clinical use.

## 1. Introduction

Systemic lupus erythematosus (SLE) is an autoimmune systemic disease that affects various organs in the body. The disease is complex with heterogenous manifestations, and both genetic and environmental factors such as ultraviolet radiation, viral infections, and exposure to certain chemicals have been implicated in disease etiology. Characteristic of this disease are autoantibodies against DNA and an increased production of type I interferons (IFNs). The innate immune system is the first responder to infections and damage and is responsible for the interferon response. Most cells can produce type I IFNs. However, plasmacytoid dendritic cells (pDCs) are the most potent producers of these cytokines. In contrast, antibody production involves the adaptive immune response. Cells such as dendritic cells (DCs), T cells, and B cells are the main players in this response.

Several anti-nuclear autoantibodies (ANAs), such as anti-double-stranded DNA (dsDNA), anti-nucleosome (nuc), anti-Sm, anti-small nuclear riboprotein (snRNP), anti-Sjogrens syndrome antigen A (SSA/Ro), anti-Sjogrens syndrome antigen B (SSB/La), anti-phospholipid (PL), and anti-C1q antibodies have been implicated in SLE, but anti-dsDNA and anti-Sm are the only antibodies that are considered specific for SLE [1]. Immunoglobulin isotypes such as IgA, IgM, IgG, IgG1, igG2, IgG3, and IgG4 have all been observed in SLE, but only the IgG isotype is used in the classification criteria for diagnosis (reviewed in [1,2]).

Autoantibodies cause inflammation and may form immune complexes that can be deposited in organs and cause local inflammation and organ damage. In SLE, the kidneys are often affected, and patients may develop lupus nephritis (LN), which is a serious complication that can lead to kidney failure [3]. The predominance of nucleic acid-associated autoantigens in SLE is noteworthy and is probably due to the ability of these antigens to also bind to members of the toll-like receptor (TLR) family of pattern recognition receptors. Other nucleic acid sensors such as cytosolic dsRNA sensors, including melanoma differentiation-associated protein 5 (MDA5) and retinoic acid-inducible gene I (RIG-1), and DNA sensors such as cyclic GMP-AMP synthase (cGAS), interferon-gamma inducible 16 (IFI16), absent in melanoma 2 (AIM2), and DNA-dependent activator of IRFs (DAI) may also be involved in SLE.

**Figure 1:**
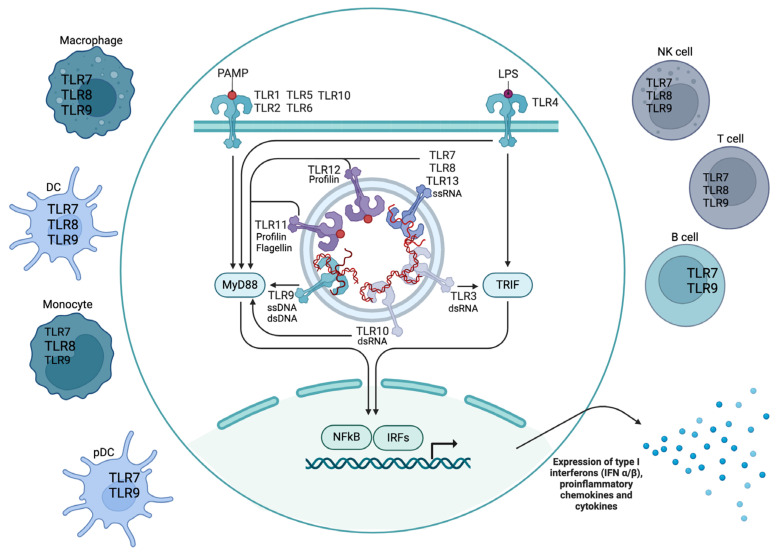
TLR signaling and expression in immune cells. TLRs are a class of proteins that are involved in immune responses upon recognizing molecules such as pathogen-associated molecular patterns (PAMPs), lipopolysaccharide (LPS), profilin, flagellin, ssDNA, dsDNA, ssRNA, and dsRNA. TLR1-TLR13 is found in mice, and TLR1-TLR10 is found in humans. The signaling pathways downstream of TLR activation are complex but can be roughly divided into MyD88-dependent and TIR domain-containing adapter-inducing IFN-β (TRIF)-dependent pathways. The myeloid differentiation factor 88 (MyD88)-dependent pathway is utilized by all TLRs except TLR3, while TLR4 can activate both pathways. Nuclear factor-kB (NFkB) comprises a family of transcription factors regulating genes involved in immune and inflammatory responses. Interferon regulatory factors (IRFs) regulate the transcription of interferons (IFNs), primarily type I IFNs. In the figure, the size of the TLR7-TLR9 letters in the different immune cells indicate their expression levels in these cells. Created with BioRender.com. DC, dendritic cell; pDC, plasmacytoid dendritic cell; NK, natural killer cell; ss, single-stranded; ds, double-stranded; IFN, interferon.

TLRs are involved in shaping the immune response by recognizing pathogen-associated molecular patterns (PAMPs) and damage-associated molecular patterns (DAMPs). Several TLRs have been identified, including TLR1-TLR10 in humans, and TLR1-TLR13 in mice. However, the *Tlr10* gene is not functional in mice [4]. TLR1–TLR2, TLR2–TLR6, and possibly TLR2–TLR10 form heterodimers [5]. The TLRs can be divided by their localization in the cell, either on the cell surface or in endosomes, and by which ligand they bind (Figure 1).

Due to their diversity, TLRs can bind to diverse ligands, making them important sensors of environmental stimuli such as bacterial and viral infections. In addition, other cellular pathways may interact with the TLR pathways in an autoimmune setting. For example, both MDA5 and cGAS were upregulated in in vitro maturated splenic B cells from a TLR7 agonist-induced lupus model, while the MDA5 pathway was also activated without additional stimulation with CD40L [6].

Upon ligand binding, TLRs undergo conformational changes leading to the recruitment of adaptor proteins such as myeloid differentiation factor 88 (MyD88) and TIR domain-containing adapter-inducing IFN-β (TRIF). This recruitment initiates downstream signaling cascades, ultimately resulting in the activation of transcription factors like nuclear factor-kB (NFkB) and interferon regulatory factors (IRFs). These transcription factors induce the expression of inflammatory chemokines, cytokines, type I interferons (IFNs), and antimicrobial peptides [5]. Due to their role in sensing and regulating immune reactions, TLRs can have various implications in autoimmunity and SLE.

In this review, we focus on TLRs’ role in human SLE and SLE mouse models, and their possible involvement in tolerance breakage. TLRs are expressed by different immune cells involved both in the innate and adaptive immune system, as well as platelets and epithelial cells. TLRs are well-known for their central role in autoimmunity and SLE.

## 2. Animal Mouse Models of SLE and LN and the Involvement of TLRs

Existing spontaneous mouse models of SLE and LN have been extensively reviewed during the past 10 years [7,8,9,10,11,12]. In addition, several genetically modified mice or other models with a lupus-like disease have been studied to determine the mechanism of SLE and LN in mice [7]. The genetically modified mouse models include knockout (KO), knock-in (KI), knock-down (KD), and transgenic (tg) mouse models.

### 2.1. Spontaneous Mouse Models of SLE and LN

The most common spontaneous mouse models used in research on the development of SLE and LN include the strains (NZBxNZW)F1 (NZBW), MRL/MpJ-Fas^lpr^ (MRL/lpr), BXSB/Yaa, and several congenic strains [13,14]. The MRL/lpr model contains a spontaneous lymphoproliferation (lpr) mutation caused by an alteration in the *Fas* gene causing a defect in FAS signaling and reduced cell death leading to lymphadenopathy. Several genetic modifications of this lupus model have been used to study the impact of TLRs on the development of autoimmune disease and are discussed later. The NZBW model, a hybrid of a New Zealand black (NZB) and a New Zealand white (NZW) mouse, developed a kidney disease resembling human LN with the development of glomerulonephritis [13,14]. Both NZB and NZW mice carry genes and develop an immunological phenotype with increased autoantibody production. However, it is only the hybrid that developed proteinuria. Several other recombinant inbred strains with an NZB or NZW background, called New Zealand mixed (NZM), have been developed to study the genetic background of murine lupus [15]. In addition, the crossing of NZB mice with several clinically normal mouse strains like SWR (SNF1 model) or SJL (NSF1 model), and the crossing of NZW with BXSB (WBF1 model), led to development of a clinical disease similar or milder to the NZBW model (reviewed in [11]). Common for most of the models is the production of autoantibodies to dsDNA and active proliferative nephritis, with a few exemptions like mild proliferative nephritis in SNF1 and WBF1 mice.

Other spontaneous mouse models include BXSB, BXD2, and (SWRxSJL)F1 mice that develop anti-dsDNA antibodies and Sm/U1snRNP antibodies. The (SWRxSJL)F1 mice may develop proteinuria at 20 weeks old with increased levels of IgG and IgA [16]. The BXSB and BXD2 models are recombinant inbreds derived from a mix of SB/Le and DBA/2J males with C57BL/6 (B6) females, respectively [17]. However, the BXSB model is unique as it mainly affects the males because of the presence of the Y-chromosome-linked autoimmune accelerator (*Yaa*). Mice with the *Yaa* locus have a duplication of the *Tlr7* gene. The *Yaa* is also important for the autoimmune phenotype in the Fc gamma receptor 2B (*FcgrIIB*)^−/−Yaa^ mice [18]. The mice develop a more severe disease than *FcgrIIB*^−/−^ without the Yaa, but with less ANA production [18]. In addition, *FcgrIIb*^−/−^ mice on a B6 background develop spontaneous and fatal glomerulonephritis [19]. The 564Igi strain (B6.129S4(Cg)-Igktm1(Igk564)Tik Ightm1(Igh564)Tik/J)) mice have heavy and light chain genes encoding the 564 immunoglobulin (derived from an autoimmune SWRxNZB hybridoma) targeted to the heavy and light chain loci of C57BL/6 mice [20]. These mice produce anti-RNA antibodies.

### 2.2. Other Genetic Mice Models Mimicking the Pathogenesis of SLE and LN

The development of *Tlr7* tg mice confirmed that the *Yaa* gene is essential for developing an autoimmune phenotype in some spontaneous models of lupus [21]. The protein tyrosine phosphatase nonreceptor 22 (PTPN22) gene encodes the lymphoid tyrosine phosphatase (LYP) in humans, and the PEST domain-enriched tyrosine phosphatase (PEP) is the homologue in mice. The LYP protein is important in regulating the function of adaptive immunity cells, and polymorphism in this gene is associated with several autoimmune diseases including SLE [22]. PTPN22 expression in myeloid cells is important for regulation of multiple pattern recognition receptors [23]. Mouse models of *Pep* KO, *Pep* KI, *Pep* KD, or *Pep* tg mice show varying degrees of autoimmunity, and this is based on the selected strain used (reviewed in [24]).

DNase1l3 deficiency has been shown in both MRL/lpr and NZBW mice, as both strains are homozygous for a missense of this enzyme in the macrophages [25]. In a recent study of conditional knockout of *Dnase1l3* in macrophages, autoantibody production and mild kidney affection were observed [26]. Systemic KO models of *Dnase1l3* on either B6 or 129SvEv backgrounds induce ANAs, specifically anti-dsDNA antibodies in addition to anti-chromatin antibodies [27,28]. A double KO of *Dnase1l3*- and *FcgrIIb*-deficient mice showed early production of anti-dsDNA antibodies [28], while *Siglecg*^−/−^ x *Dnase1l3*^−/−^ double KO mice, but not *Siglecg*^−/−^ x *Dnase1*^−/−^ KO mice, produced autoantibodies only later in life [29]. However, *Dnase1*^−/−^ mice produced ANAs, had glomerular immune complex deposits, and developed glomerulonephritis, demonstrating the importance of chromatin degradation to maintain tolerance against nuclear antigens [30,31].

LYN is an Src kinase associated with SLE. *Lyn*^−/−^ mice showed increased levels of IgG and immune complex deposition and developed glomerulonephritis [32]. Other SLE symptoms included anemia, leukopenia, and thrombocytopenia. LYN is an inhibitor of IRF5 and thereby regulates signaling through TLRs [33]. Mice with a B-cell-specific deletion of CR6-interacting factor 1 (CRIF1), a nuclear transcriptional regulator and a mitochondrial inner membrane protein, had a lupus-like phenotype with anti-dsDNA antibody production and development of LN [34]. Depletion of CRIF1 has been shown to enhance activation trough TLR7 and TLR9 [35]. Deficiency in B-lymphocyte-induced maturation protein (BLIMP)-1 in dendritic cells (DCs) (*Prdm1*) led to a lupus-like phenotype with increased subsets of T follicular helper (Tfh) cells and plasma cells [36,37,38]. Another study showed that Blimp-1 directly suppressed interleukin-1 receptor-associated kinase 3 (*Irak3*) [39]. In a recent KI tg model, the gene *ERN1* encoding inositol-requiring enzyme 1α (IRE1α) carrying a heterozygous mutation led to a defect in IRE1α ribonuclease activity on X-box binding protein 1 (XBP1) splicing; the mice developed a broad panel of autoantibodies including antibodies against chromatin, Scl-100, or Sm/RNP [40].

Transcription factor E2F2-deficient mice with a mixed 129/sv x B6 background showed diffuse late onset SLE with systemic inflammatory infiltrates in the lung and liver, splenomegaly, immune complex deposition, and varying anti-dsDNA antibody titers [41]. However, backcrossing the original *E2f2*^−/−^ mice into a pure B6 background eliminated the autoimmunity. Introducing an overexpression of the anti-apoptotic Bcl-2 protein in the B cells of these mice induced increased anti-DNA antibodies and development of mild glomerulonephritis [42]. Another study showed that E2F2 directly regulated the expression of MyD88, the adaptor of most of the TLRs, by binding to its promoter [43]. Phospholipase D family member 4 (PLD4) mutant mice (BALB/c *Pld4* thss/thss) developed anti-dsDNA and ANAs [44,45]. PLD4 is a 5′ exonuclease important for the degradation of single-stranded (ss) DNA in the endolysosomes, regulating ssDNA signaling through TLRs [46]. A gain-of-function mutation of another phospholipase Cγ2 (PLCγ2) in mice leads to an autoimmune phenotype [47].

Wiskott–Aldrich Syndrome (WAS) is a rare disease that is caused by WAS protein (WASP) deficiency and is characterized by diverse immune aberrations, including the production of autoantibodies. A mouse model has been developed where B cells, but not any other hematopoietic lineages, fail to express WASP [48]. This model has been termed B^WAS−/−^, and the mice develop high titers of anti-DNA and anti-RNA antibodies [49]. The mechanism behind development of autoimmunity in this model has been related to the hyperresponsiveness of *WAS*^−/−^ B cells to both BCR and TLR signals.

**Figure 2:**
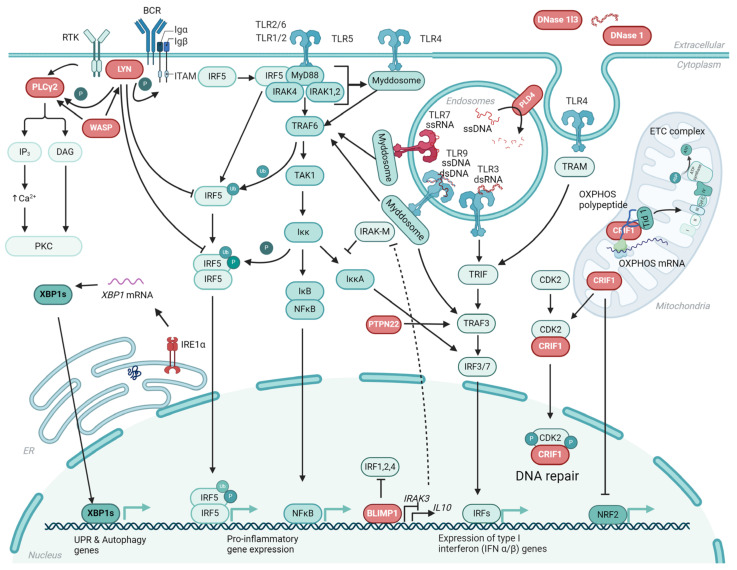
Overview of single-gene knockout (KO), knock-in (KI), or mutations in mice causing a lupus-like phenotype by influencing toll-like receptor (TLR) signaling and gene expression. Normal signaling pathways are shown with single-gene KO or mutations marked in red. *Tlr7* duplications in both spontaneous lupus models carrying the *Yaa* gene and in *TLR7* transgenic mice induce spontaneous autoimmunity. *Dnase1l3* and *Dnase1* KO mice have reduced clearance of circulating chromatin, thus increasing the antigens for TLRs. PLD4 mutant mice have increased signaling through TLRs due to reduced degradation of ssDNA in endosomes. CRIF1 deficiency influences CDK2-induced DNA repair, NRF2 binding, and formation of the ETC complex [50]. BLIMP1 normally controls the binding of IRF1, 2, and 4 and increases IL-10 expression. It also suppresses the expression of IRAK3 an inhibitor of IRF7 signaling [51]. PTPN22 inhibits various signaling pathways but acts as a selective promoter of type I interferon by promoting autoubiquitination of TRAF3 and phosphorylation of IRF3 and IRF7 [23]. LYN phosphorylates ITAM and PLCγ2 and inhibits IRF5 activation [52]. PLCγ2 activation via tyrosine kinases like LYN leads to increased Ca^2+^ signaling and a gain-of-function mutation, which was shown to cause hyperreactive external calcium entry [53]. WASP affects many parts of the BCR signaling pathways [54] and B-cell-specific WAS deficient mice develop autoantibodies against both DNA and RNA [48]. IRE1α is an ER membrane protein important for transducing signals of misfolded protein accumulation in ER to the nucleus by splicing X-box binding 1 (*XBP1*) mRNA and leading to the production of stable transcription factor XBP1 (XBP1s) [55]. XBP1s targets various genes involved in multiple cellular functions [56]. Created with BioRender.com. KI, knock-in; KO, knockout; PLD4, phospholipase D family member 4; UPR, unfolded protein response; ssDNA, single-stranded DNA; ETC, electron transport chain; BCR, B-cell receptor; WAS, Wiskott–Aldrich syndrome; ER, endoplasmatic reticulum; IRF, interferon regulatory factors; CRIF, CR6-interacting factor 1; IRAK, interleukin-1 receptor-associated kinase; PTPN, protein tyrosine phosphatase nonreceptor 22; XBP1, X-box binding 1; PLCγ2, phospholipase Cγ2; BLIMP, B-lymphocyte-induced maturation protein.

Taken together, there are several different genes that are either directly or indirectly linked to TLR signaling and are thus important for immune homeostasis. Figure 2 summarizes some of the most central genes and gene products linked to TLR signaling that may cause a lupus-like phenotype if mutated or knocked out.

### 2.3. Inducible SLE Mouse Models

Pristane (2,6,10,14 tetramethylpentadecane) has been used since the 1980s as an inducible model of LN in various healthy mice strains like BALB/c, SJL/J, and C57BL/6, as it results in immune complex-mediated glomerulonephritis (reviewed in [57]). It is also a good model for SLE in general since the mice may develop erosive arthritis, skin rash and, in more severe cases, pulmonary vasculitis and haemorrhage [58]. However, the choice of strain used is important as they show huge differences in their autoantibody profiles [59]. In addition, pristane may induce ANAs, anti-dsDNA, and anti-SnRNP antibodies and show an overproduction of type I IFN, which makes it very suitable as a model for SLE since high amounts of type I IFN are observed in 50% of SLE patients [60]. The model relies on the expression of TLR7 [61] and has been used to determine the role of other TLRs like TLR2 [62], TLR4 [63], and TLR9 [63,64], in addition to induction factors like BAFF [65] and tonicity-responsive enhancer-binding protein (TonEBP) [66] and signaling molecules and sensors like IRF7 [67], cGAS-STING pathway [68], and IRAK4 [69] in murine lupus. Pristane has also been used to accelerate the disease in NZBW and the SNF1 model [70,71]. Using pristane treatment in normal B6 and B6/lpr and B6/gld mice demonstrated the contribution of defects in the Fas or Fas ligand [72].

A newer inducible lupus model involved topical treatment with resiquimod or imiquimod creams containing TLR-7/8 or TLR7 ligand/agonist in wild-type (WT) mice. When applied three times a week for 4-8 weeks, it induced anti-dsDNA antibodies, glomerulonephritis, hepatitis, carditis, and photosensitivity in these mice [73]. The application of imiquimod to the skin is a prerequisite for inducing the disease, as oral administration and injection of imiquimod do not lead to the same immune cell activation. In graft-versus-host disease (GVHD) and chronic GVHD (cGVHD), donor lymphocytes are injected into a semi-allogenic recipient to induce a lupus-like syndrome [74]. Autoantibody-mediated (lupus-like) cGVHD in mice is caused by alloantibody secretion and deposition, in addition to B- and T-cell infiltrations in the affected tissues [75]. A recent study showed increased expression of TLR7 in mice with cGVHD [76]. Garimella et al. (2021) used syngeneic apoptotic cells to break B-cell tolerance in C57Bl and UNC93B1 mutant mice that lacked signaling through TLR3, TLR7, and TLR9 [77]. They found reduced responses against known autoantigens in the mutant mice, showing the importance of endosomal TLR in tolerance breakage against lupus autoantigens.

### 2.4. Acceleration of Spontaneous Lupus Models and Humanized Mouse

Some of the spontaneous models develop SLE and LN over a long time (5–12 months) and the disease manifestations are very heterogenic with some mice never developing proteinuria, making the models difficult to use in treatment strategies to prevent LN. To solve this, several different compounds have been used to study the mechanism of SLE by accelerating different processes in spontaneous mouse models. This has also included the use of pristane and imiquimod, accelerating the development of proteinuria in NZBW and MRL/lpr mice [78]. Recently, resiquimod treatment of B6.Sle1.Sle2.Sle3 triple congenic mice induced an increased leaky gut, and this was shown to be due to TLR7/8 activation [79]. Other compounds normally not inducing SLE in healthy mice include poly IC [80], IFNα [81,82], mercury [81,82], respirable crystalline silica dust particles [83], LPS [83], and CpG [84] and these have also been used for this purpose.

Humanized mouse models of SLE involve transferring PBMC from SLE patients to immunodeficient mice or transferring human hematopoietic stems cells to immunodeficient mice with subsequent induction of lupus via intraperitoneal injection of pristane (reviewed in [85]). Other studies have introduced human genes into mice strains, like human *TLR8* in an SLE1.Yaa strain that induced fatal anemia [86]. In a recent study by Cakan et al. (2023) to study the role of TLR7 and TLR9 in induction of B-cell tolerance, they used NOD-scid-common gamma chain (γc) knockout (NSG) immunodeficient mice with CD34^+^ human fetal hematopoietic stem cells (HSCs) transduced with GFP-tagged lentivirus expressing shRNA to inhibit the expression of MYD88, TLR7, and TLR9 [87]. It was shown that TLR9 is important for maintaining central B-cell tolerance, as both TLR9 and MYD88 silencing resulted in increased polyreactive or ANA-producing B cells. In addition, the study demonstrated that CXCL4 production sequestered TLR9 ligands away from the late endosomes and thus inhibited TLR9 function in B cells.

## *3.* TLR Signaling in SLE—An Update on Recent Findings

TLRs may contribute to the chronic activation of the immune system in patients with SLE or in lupus-prone or -induced mice. High mobility group box 1 (HMGB1) is a typical DAMP and can be released by apoptotic and necrotic cells. Higher levels of serum HMGB1 and anti-HMGB1 antibodies correlating with disease activity have been found in SLE patients [88,89,90,91]. HMGB1 can be recognized by TLR2, TLR4, and TLR5 [92,93]. Ma et al. (2018) identified TLR4^+^CXCR4^+^ plasma cells in peripheral blood and kidney tissue, correlating with anti-dsDNA levels in SLE patients and lupus-prone mice, and showed that TLR4 blockade in vitro reduced anti-dsDNA IgG secretion from these cells [94]. Also, in MRL/lpr mice, the expression of TLR4^+^CXCR4^+^ plasma cells was significantly increased. Interestingly, this cell population decreased upon Nrf2 overexpression [95], indicating a potential role in LN disease progression and revealing this pathway as a possible target for treatment. An investigation into the expression and interplay of HMGB1 and *TLR4* in patients with neuropsychiatric SLE (NPSLE), found either protein or mRNA expression to be increased in serum and PBMCs, respectively, but did not observe significant correlation between HMGB1 and *TLR4* expression and NPSLE-related seizures [96]. Several studies have also investigated the genetic association between *TLR2* and *TLR4* polymorphisms and SLE susceptibility [97,98,99]. However, the results from those studies have not provided evidence for *TLR2* and *TLR4* gene polymorphisms and SLE.

TLR5 recognizes bacterial flagellin. A recent study by Alajoleen et al. (2024) using TLR5-deficient MRL/lpr mice demonstrated a worsening of the disease, possibly due to an increased germinal center reaction and suppression of regulatory lymphocytes [100]. There have been very few studies on the role of TLR5 in SLE and LN. Both increased and decreased levels of TLR5 expression have been shown in different organs during murine lupus disease progression [101]. In addition, several studies on polymorphisms in SLE have indicated no association with *TLR5* polymorphisms even though an increase in *TLR5* gene expression was observed in LN biopsies [98,102,103]. However, Hou et al. (2023) recently identified a mutation in TLR5 in early-onset pediatric SLE with renal, hematological, and central nervous system involvement [104]. The new findings indicate that TLR5 influences important regulatory functions of the immune system, and more studies on its role in autoimmune diseases are required.

Among the TLRs, TLR3, TLR7, TLR8, TLR9, TLR10, and TLR13 (Figure 1) are specific for nucleic acids and perhaps most relevant to SLE. TLR3 recognizes dsRNA, while TLR7 and TLR8 recognize ssRNA. TLR9 identifies unmethylated CpG DNA. TLR13 is specific for rRNA regions, particularly certain 23S rRNA motifs found in bacteria [105,106]. Human TLR10 has been shown to bind to dsRNA in vitro at acidic pH, suggesting it has an endosomal location [107] in addition to having a plasma membrane localization [108]. The exact mechanisms of TLR10 are somewhat unclear and it is suspected to have both pro- and anti-inflammatory properties (reviewed in [108]). Interestingly, in relation to SLE, Lee et al. (2018) showed that the binding of TLR10 to dsRNA activated the MyD88 signaling pathway and suppression of IRF7-dependent type I IFN expression as well as inhibition of TLR3 signaling through sequestering dsRNA from this receptor [107]. Engagement of TLRs is important in the pathogenesis of SLE, contributing to the production of type I IFNs and the activation of autoreactive B cells (reviewed in [109]).

Platelets also express TLRs. In SLE, platelets are activated, and their abnormal expression in blood can mirror disease activity. Platelets express FcγRs and TLRs TLR1-TLR4, TLR7, and TLR9 [110], indicating that different PAMPs, DAMPs, immune complexes, and nucleic acids can activate platelets (reviewed in [110,111,112,113]). When activated, platelets express CD40L and P-selectin on the cell surface, contributing to interaction with immune cells [113]. In addition, activated platelets release extracellular vesicles, leading to constituents such as HMGB1 and P-selectin being accessible to other cells not normally in contact with these components [110,111,112,113]. Activated platelets and extracellular vesicles can stimulate neutrophils to undergo neutrophil extracellular trap (NET)osis, pDCs to produce IFNα, B cells to produce autoantibodies, regulatory T cells to downregulate FOXP3, and maturation of monocytes to APCs [111], all factors that can contribute to disease progression in SLE. A recent study by Baroni Pietto et al. (2024) showed that platelets could contribute to inflammation in SLE patients [114], and similar findings are reviewed in [111]. Interestingly, Tay et al. (2024) found TLR7 expression in platelets to be important for platelet–low-density neutrophil (LDN) complexes. LDNs are a subset of neutrophils associated with SLE, while platelet-neutrophil complexes have been observed after platelet activation and are formed during inflammation.

The nucleic acid-sensing TLRs TLR7, TLR8, and TLR9 (Figure 1) have been well studied in relation to SLE. All three are located intracellularly in endosomes, but their expression varies between different subsets of immune cells, which in turn affects how they are implicated in SLE. T cells and natural killer cells express low amounts of all of them, while B cells and pDCs express both TLR7 and TLR9 [115]. Monocytes express low levels of TLR7 and TLR9, but instead express TLR8, which is absent in B cells and pDCs. DCs and macrophages express TLR7, TLR8, and TLR9 [116,117]. TLR7 and TLR9 have homologous ligands and functions in mice and humans, whereas TLR8 is not bound by ssRNA in mice and its murine ligand is yet to be identified [118]. For a while, it was thought that TLR8 may not be functional in mice. For this reason, many of the studies on TLR function in SLE have focused on TLR7 and TLR9. Still, it has been demonstrated that murine TLR8 could have regulatory functions that may be independent of ligand binding [116].

### 3.1. TLR Driven Autoantibody Production and Tolerance Breakage

**Figure 3:**
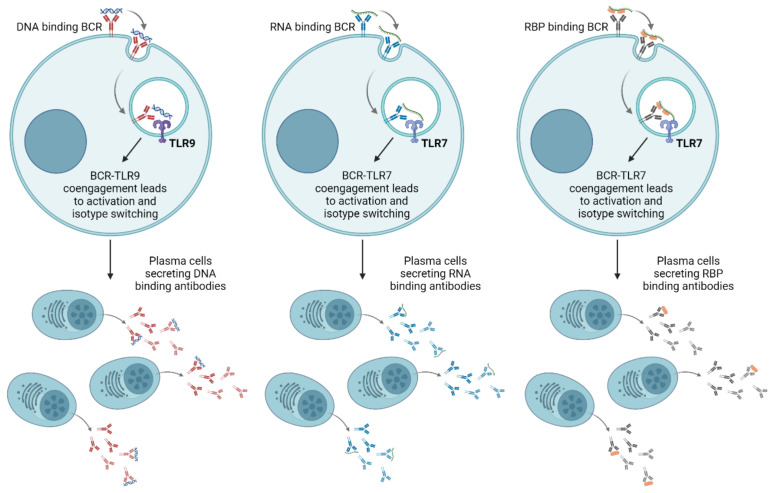
TLR-driven autoantibody production. Recognition of extracellular nucleic acids or proteins bound to nucleic acids by naïve B cells via the B-cell receptor (BCR) causes internalization of the BCR–antigen complex, which ends up in an endosome. Endosomal toll-like receptors (TLRs) like TLR7 and TLR9 may then also encounter the internalized nucleic acid-containing antigens. Co-engagement of BCR and TLR via antigens can lead to activation of the B cell and induce isotype switching to IgG. Consequently, a large number of autoantibody secreting plasma cells can be generated. Created with BioRender.com. RBP, RNA-binding protein.

In addition to stimulating a general inflammatory response by causing the production of inflammatory cytokines, TLRs can also be directly involved in the production of autoantibodies. An accepted description of how this may occur is through a specific form of T-independent B-cell activation where self-DNA or self-RNA binds to the BCR of a naïve B cell that expresses a BCR specific to DNA or RNA (Figure 3) [119,120]. This leads to internalization of the BCR-DNA/RNA complex in the endosome, which may fuse with another endosome containing TLRs. In this way, the internalized DNA or RNA may also bind to and activate TLR9 or TLR7, respectively. It has been demonstrated that this co-engagement of the BCR and a TLR is enough to activate B cells without help from T cells, in turn leading to the maturation of plasma cells producing antibodies that bind to DNA or RNA [121,122,123,124,125]. Likewise, proteins bound to DNA or RNA may also be internalized via binding to a BCR that is specific for that protein and bring with them DNA or RNA into an endosome, activating TLRs and causing the maturation of plasma cells producing autoantibodies like anti-Sm, anti-RNP, and anti-nucleosome. Normally, T-independent B-cell activation results only in the production of IgM antibodies. However, CpG binding to TLR9 on B cells can also activate antibody class switching to the Th1-like isotypes IgG2a, IgG2b, and IgG3, which are commonly seen in SLE [126,127]. One study even found that class switching of anti-DNA antibodies to IgG2a and IgG2b isotypes was impaired in TLR9-deficient mice [128]. Several studies have demonstrated the concept of TLR-BCR co-engagement, for instance by immunizing mice with protein antigens linked to CpG DNA, chromatin-containing immune complexes, or RNA-containing immune complexes, with subsequently enhanced production of antigen-specific antibodies [122,125,129]. They also confirmed the involvement of TLR9 or MyD88 in these results by knocking out or inhibiting them. This was recently confirmed by Cakan et al. (2023), as described above [87]. Furthermore, a recent study on the same concept investigating TLR4 and TLR5 also demonstrated that B-cell activation mediated by TLR-BCR co-engagement is T-cell independent, through performing similar studies using a mouse model which was devoid of T cells [130].

Upon the initial discovery of TLR9, it was stated that TLR9 was able to discriminate between bacterial DNA and self-DNA because its ligand, unmethylated CpG DNA, is quite scarce in mammalians. However, unmethylated CpG DNA does exist in mammalian DNA as well, and several studies have demonstrated a dependence on TLR9 signaling to produce anti-DNA antibodies associated with SLE [49,124,131]. A more accepted notion today is that the intracellular location of the nucleic acid-sensing TLRs is the main mechanism of discriminating self from non-self. Indeed, since B cells generally do not endocytose extracellular material unless the BCR is bound, host-derived DNA or RNA, for instance originating from dead cells, would not normally come into contact with TLR9 or TLR7 in B cells and therefore would not activate them. In contrast, B cells carrying a nucleic acid-binding BCR naturally endocytose nucleic acids, possibly breaking self-tolerance in these cells [131]. Moreover, engagement of TLR9 can protect B cells from spontaneous or BCR-mediated apoptosis, contributing to tolerance breakage [132,133,134]. Further supporting the notion that TLR-BCR dual engagement is a key mechanism for the maturation of plasma cells producing nucleic acid-specific antibodies is the fact that global or B-cell-specific deletion of MyD88 in lupus-prone mice has been shown to suppress the production of all antinuclear antibodies [125,135,136,137]. More specifically, several studies have demonstrated that the specific deletion of TLR7 or TLR9, either globally or in B cells only, abrogated production of anti-RNA and anti-DNA antibodies, respectively [49,117,124,131,135,138].

### 3.2. Diverse Effects of Different TLRs on SLE Pathogenesis—TLR7 As the Main Driver of Disease

Despite the direct influence of both TLR7 and TLR9 on autoantibody production, as well as the fact that TLR7, TLR8, and TLR9 all activate the same signaling pathways, their roles in the pathogenesis of SLE are not equivalent. Numerous studies have implicated TLR7 as the main driver of SLE disease, while both TLR8 and TLR9 have been shown to have more regulatory roles where they contribute to dampening TLR7 signaling and thereby prevent autoimmunity [116,124,139]. This is demonstrated by the fact that knocking out either *Tlr8* or *Tlr9* in healthy C57BL/6 mice induced SLE-like autoimmune disease, while additional knockout of *Tlr7* eliminated disease symptoms, indicating that the disease development was dependent on TLR7 [116,140]. Single knockout of *Tlr7* in lupus-prone mice also ameliorated disease [124]. Moreover, gene duplication of *Tlr7*, as seen in mice bearing the Y-linked autoimmune accelerator (*Yaa*) locus, contributed to accelerating autoimmune disease. In addition, topical treatment of mice with the TLR7 agonist imiquimod also induced SLE-like disease [73].

Strong evidence supports the disease-promoting role of TLR7 in humans as well. The *Tlr7* gene is located on the X chromosome and the risk of developing SLE correlates with the number of X chromosomes an individual carries, demonstrated by the female predominance and increased incidence in men with Klinefelter syndrome (47, XXY) [141]. X-chromosome inactivation normally contributes to the silencing of one arbitrary X chromosome, but not all genes are affected, and it has been shown that *Tlr7* escapes X-chromosome inactivation in B cells, monocytes, and pDCs in both women and Klinefelter syndrome men [142]. Recently, it was demonstrated that the gene encoding TLR8, which is closely located to *Tlr7*, also escapes X-chromosome inactivation in immune cells in women and Klinefelter syndrome men [143]. Increased expression of TLR7, independent of gene copy number, has also been associated with more severe SLE disease in humans [144]. A specific *Tlr7* polymorphism (rs3853839-G) has been demonstrated to cause increased expression of TLR7 and is associated with SLE in humans [145,146,147]. Recently, a never-before-seen *Tlr7* gain-of-function gene variant (*Tlr7^Y264H^*) was identified in a young girl suffering from SLE [148]. This variant of TLR7 was shown to have increased affinity to guanosine present in RNA and enhanced NF-κB activation. When introduced into C57BL/6 mice, the *Tlr7^Y264H^* gene induced an SLE-like disease.

### 3.3. Regulatory Functions of TLR8 and TLR9

As previously mentioned, global knockout of *Tlr9* induces or worsens lupus-like disease in several mouse models. This concept has been demonstrated in a number of different mouse models, including MRL/lpr, MRL/+, B6-lpr/lpr, B6.Nba2, FcγRIIB^−/−^, Plcg2^Ali5/+^, and pristane-treated BALB/c [64,124,149,150,151,152,153]. Similar to TLR9, TLR8 has also been implicated to have regulatory functions on TLR7 [116]. Thus, C57BL/6 mice who are deficient in both *Tlr8* and *Tlr9* suffered from more pronounced disease compared with mice lacking only one of these genes [140]. However, the same study demonstrated that TLR8 and TLR9 exerted their regulatory effects in different cell types. TLR8 seemed to mainly act in DCs, whereas TLR9 mainly exerted its regulatory functions in B cells [140].

An extensive amount of work has been conducted to study the relationship between TLR7 and TLR9 in B cells. It has been demonstrated that B-cell-specific knockout of *Tlr7* is enough to ameliorate disease in lupus-prone mice, while B-cell-specific knockout of *Tlr9* exacerbates disease [49,117]. One study showed that absence of TLR9 in B cells caused exacerbated nephritis, while overexpression of TLR9 in B cells caused reduction of both nephritis and proteinuria [117]. The latter was demonstrated in both MRL/lpr and FcγRIIB^−/−^.Yaa mice [117]. In contrast, deletion of TLR9 in cDCs, pDCs, macrophages, or neutrophils had no effect on SLE disease parameters, further supporting the notion that it is the B-cell-intrinsic TLR9 which is protective in SLE. In line with this, deletion of TLR7 in CD11c^+^ cell populations mainly comprising DCs had no impact on SLE disease parameters in MRL/lpr mice [154]. However, B-cell-specific deletion of TLR7 did ameliorate disease, especially in TLR9-deficient mice.

Since exacerbation of SLE disease in *Tlr9*^−/−^ mice depends on TLR7, it has been hypothesized that TLR9, either directly or indirectly, negatively regulates TLR7 activity. In that case, deletion of TLR9 would increase TLR7 activity and, in turn, cause worsened disease. In line with this, several studies have demonstrated higher expression of TLR7 and increased response to TLR7 ligands in *Tlr9* knockout models [140,151,155]. For instance, B cells from *Tlr9*^−/−^ and *Tlr8*^−/−^*Tlr9*^−/−^ C57BL/6 mice responded more strongly to the TLR7 ligand R848 than B cells from WT or *Tlr8*^−/−^ mice [140]. B cells from *Tlr9*^−/−^ B6.Nba2.Yaa mice also responded more strongly to imiquimod and expressed higher levels of TLR7 than B cells from *Tlr9*^+/+^ mice [151].

One popular hypothesis explaining how TLR9 may indirectly regulate TLR7 activity suggests that deletion of TLR9 causes increased trafficking of TLR7 to late endosomes because TLR7 and TLR9 compete for the same shuttle mechanism [156]. UNC93B1 is an endoplasmatic reticulum (ER)-resident chaperone that controls trafficking of nucleic acid-sensing TLRs as well as TLR5, TLR11, and TLR12 from the ER to their respective locations in endosomes or on the cell surface [157,158]. Upon viral infection or TLR signaling, nucleic acid-sensing TLRs are transported to endosomes. TLR7 and TLR9 both bind to UNC93B1, which has the strongest affinity for TLR9 [159]. However, a mutation in UNC93B1 (D34A) causes enhanced affinity for TLR7 and, thus, enhanced trafficking of TLR7 to endolysosomes, which in turn induces TLR7-dependent systemic inflammation [156]. Based on these findings, it has been suggested that when TLR9 is absent, this causes less competition for binding to UNC93B1 and, thus, increased trafficking of TLR7 to endosomes, which could be the mechanism that drives the worsened disease seen in *Tlr9*^−/−^ mice [156]. The same hypothesis has also been proposed for *Tlr8*^−/−^ mice, as TLR8 is also shuttled to endosomes by UNC93B1 [140]. However, this theory has recently been challenged as it was demonstrated that the localization of TLR7 was the same in WT and *Tlr9*^−/−^ mice, probably because the mere absence of TLR9 (or TLR8) does not increase the affinity of UNC93B1 for TLR7 [160]. TLR7 and TLR9 were also largely located in separate compartments, indicating that they should not compete with each other for binding to UNC93B1. Interestingly, unlike previously mentioned findings, the current study did not identify differences in expression and signaling of TLR7 between WT and *Tlr9*^−/−^ mice. Instead, it was explored whether TLR9 could regulate TLR7 activity through other mechanisms. Point-mutated versions of TLR9 that lacked either ligand or MyD88 binding were expressed in MRL/lpr mice [160]. Both mutated versions of TLR9 increased survival compared with *Tlr9*^−/−^ mice, suggesting that simply the presence of TLR9, despite being “dysfunctional”, is protective. Furthermore, the TLR9 version that could bind ligand but did not signal through MyD88 was the most protective, suggesting that TLR9 has protective effects that are ligand-dependent but MyD88-independent. This also indicates that TLR9 signaling through MyD88 does promote disease, as would be expected [156].

Despite TLR8 not being so well studied as TLR9, several studies support the notion that TLR8 also has regulatory functions on TLR7. For instance, TLR8-deficient C57BL/6 mice showed increased expression of TLR7 in DCs, which was accompanied by increased responses to TLR7 agonists and increased NF-κB activation [116]. These mice also had elevated levels of both anti-RNA and anti-DNA antibodies. In contrast, TLR7^−/−^ and *Tlr8*^−/−^*Tlr7*^−/−^ mice did not produce autoantibodies. The *Tlr8*^−/−^ mice also had increased numbers of plasma cells, and *Tlr8*^−/−^ DC had increased cytokine production compared with WT DCs. However, there was no difference in cytokine production by macrophages, supporting the fact that the effect of TLR8 on TLR7 is cell-type-specific. Another study, also based on C57BL/6, supported the finding that DCs express higher levels of TLR7 when TLR8 is knocked out [140]. These *Tlr8*^−/−^ DCs also responded more strongly to the TLR7 ligand R848 compared with DCs from WT mice, and the same pattern was observed for pDCs. One study found that a high-fat diet exacerbated SLE in *Tlr8* knockout mice, an effect which was dependent on TLR7 since it was abrogated in *Tlr7/8* KO mice [161]. Again, that study also found that *Tlr8* knockout mice expressed higher levels of TLR7 than WT mice in DCs as well as macrophages. Interestingly, in a human setting, a mutation in TLR8 was recently described and found to cause severe autoimmune disease in the monozygotic twins who carried it [162]. The mechanism behind the development of autoimmunity was found to be reduced ability of TLR8 to regulate TLR7 signaling, as well as increased binding of TLR8 to TLR7 ligands, which increased TLR7 signaling.

### 3.4. Regulation of TLR Signaling—Endosomal Trafficking and Glycosylation

In addition to TLR8 and TLR9 having regulatory functions affecting TLR7, several other proteins and signaling pathways can influence the levels of signaling by these TLRs in a cell (reviewed in [163]). One such protein is the previously mentioned UNC93B1, which has gained much attention during recent years and can influence TLR signaling in different ways. As mentioned, UNC93B1 is required for the trafficking of nucleic acid-sensing TLRs to endosomes. One study found that UNC93B1 must be glycosylated at a specific asparagine residue in order to recruit MyD88 and signal properly upon TLR9 activation. This glycosylation was not necessary for TLR7 signaling to function [164]. Another study identified a mutation in UNC93B1 (S282A) that abolished signaling in TLR9, but did not affect other TLRs [165]. The mutation did not alter TLR9 trafficking, but inhibited binding of TLR9 to its ligand. It was demonstrated that TLR9 needs to be released from UNC93B1 to be able to signal properly. Recently, Ni et al. (2024) showed that this release depended on the removal of a palmitoylation, initially added to TLR9 in the Golgi and necessary for its trafficking to endosomes [166]. Conversely, TLR7 does not need to be released in order to function. Indeed, another study discovered a different mutation in UNC93B1 (530-PKP/AAA-532) that caused enhanced signaling through TLR7 without affecting TLR7 trafficking [167]. Under normal conditions, the protein syntenin-1 binds to UNC93B1 after stimulation of TLR7 (but not other TLRs) and causes TLR7 to be taken up into intraluminal vesicles and exosomes, which is likely to dampen continued TLR7 signaling. K63-linked ubiquitinylation, which normally marks cargo for sorting into intraluminal vesicles, was markedly reduced in the mutated version of UNC93B1 and reduced ubiquitinylation correlated with enhanced TLR7 signaling. Phosphorylation of UNC93B1 at specific sites was required for recruitment of syntenin-1. Mice carrying the 530-PKP/AAA-532 mutation in UNC93B1 developed severe systemic inflammation and produced ANAs. However, upon knockout of *Tlr7*, the mice were rescued from disease, supporting the hypothesis that the mutation specifically affected TLR7 signaling [167]. In humans, a few different mutations in UNC93B1 that cause increased TLR7 signaling through various mechanisms have been identified in SLE patients and underscore the importance of a functional UNC93B1 [168,169].

Glycosylation of TLRs represents another way to regulate their signaling. One study using a CRISPR/Cas9 screening method identified the oligosaccharide transferase complex (OSTC) as indispensable for TLR5, TLR7, and TLR9 responses [170]. OSTC glycosylates proteins in the ER and its absence inhibits cell surface expression of TLR5. Although it has not been conclusively demonstrated, it was hypothesized that glycosylation by OSTC induces maturation and trafficking of TLR5, TLR7, and TLR9 from the ER. The activity of TLR3 has also been found to depend on glycosylation [171]. In addition, Neu1 sialidase, which cleaves sialic acid residues from glycosylated sites of TLRs, has been shown to be important for the activity of TLR2, TLR3, and TLR4 [172]. Overall, defects in glycosylation of a variety of immune cell-related proteins have been associated with SLE in both mice and humans [173].

### 3.5. Diverse Effects of TLR Signaling on Autoantibody Repertoire in Different Mouse Models of SLE

As previously mentioned, TLR7 and TLR9 have been specifically linked to the production of anti-RNA and anti-DNA antibodies, respectively. For instance, this has been shown in both the MRL/lpr mouse model and the B^WAS−/−^ model. Interestingly, in both models, deletion of *Tlr9* not only suppressed the production of anti-DNA antibodies but also increased the production of anti-RNA antibodies [49,135]. One study using the MRL/lpr mouse model found that B-cell-specific deletion of TLR9 more or less completely inhibited the production of anti-DNA antibodies, while B-cell-specific overexpression of TLR9 increased the anti-DNA-to-anti-RNA antibody ratio [117]. However, the same study did not report this effect in the FcγRIIB^−/−.^Yaa model. Indeed, results regarding the type and amount of antibodies produced in TLR knockout models vary considerably between different genetic backgrounds. For instance, *Plcg2*^Ali5/+^-*Tlr9*^−/−^ mice were shown to produce similar amounts of anti-DNA auto-antibodies as *Plcg2*^Ali5/+^-*Tlr9*^+/+^ mice, while anti-nucleosome antibodies were significantly decreased and anti-nucleolar antibodies were increased in *Tlr9*^−/−^ mice [153]. Similarly, another study also found that development of anti-nucleosome antibodies was abrogated in B6-lpr/lpr mice when TLR9 was knocked out, whereas the anti-dsDNA antibody titer was significantly higher [150]. Increased levels of anti-dsDNA antibodies and decreased levels of anti-chromatin antibodies upon knockout of *Tlr9* have also been reported in B6.Nba2.Yaa mice [151]. One study that looked at the effect of diet on SLE pathogenesis in *Tlr8*^−/−^ mice found that additional knockout of *Tlr7* significantly decreased the amount of anti-DNA antibodies, indicating that the production of anti-DNA antibodies depended on TLR7 [161]. Furthermore, treatment of mice with the TLR7 agonist imiquimod led to the production of both anti-DNA and anti-RNA antibodies, and production of anti-DNA antibodies was not abrogated by knocking out *Tlr9* in imiquimod-treated mice [73]. Taken together, these results suggest that TLR9 is not the only potential driver of anti-DNA antibody production in SLE.

### 3.6. Age-Associated B Cells as the Main Source of TLR Driven Autoantibody Production

A specific subset of B cells, referred to as age-associated B cells (ABCs), are highly dependent on TLR signaling and are also strongly associated with SLE (reviewed in [174,175]). Several studies have linked ABCs to SLE in both mice and humans, where they are thought to be the precursor cells to autoantibody-secreting cells [176]. In humans, ABCs are sometimes also referred to as atypical memory B cells or double-negative (DN) cells [174,177]. Large numbers of ABCs are found both in human SLE patients and several different murine SLE models. For instance, ABCs accumulate drastically in NZBW, MRL/lpr, *SLC*^−/−^, and *Mer*^−/−^ mice with disease onset [178,179]. Previously, it was shown that ABCs increased in individuals with enhanced TLR7 expression [148]. Differentiation of ABCs occurs upon engagement of TLR7 or TLR9, together with the BCR [175]. Dai et al. (2024), found the transcription factor ZEB2 to be essential for ABC differentiation in vitro and vital for ABC formation in TLR7-induced lupus disease, while mice deficient in *Zeb2* and *ZEB2* haploinsufficient persons had reduced numbers of ABCs [180]. In addition, cytokine signaling through IFNγ and IL-21, as well as stimulation of CD40, is necessary for differentiation of ABCs [175,177,181]. B cells that express a BCR specific for DNA, RNA, or nucleic acid-associated proteins are probably inclined to follow this differentiation program, as BCR-TLR co-engagement naturally occurs in these cells. Indeed, a recent study found that 3H9^+^ mice, whose BCRs mainly bind to nucleosomes or dsDNA, had increased numbers of ABCs compared with control animals, suggesting the ABCs originated from the DNA-binding B cells [182]. Also, when *Tlr9* was knocked out in these mice, the ABC population decreased. The same study demonstrated that ABCs are a dynamic B-cell population that can develop into plasma cells or have a more memory-like phenotype and probably go through multiple rounds of reactivation. One of the hallmarks of ABCs is expression of the T-box transcription factor (T-bet). TLR9-BCR crosslinking has been found to stimulate activation of T-bet, which is involved in class switching to IgG2a and 2b isotypes [128,183]. Interestingly, a distinct feature of ABCs is their germinal center-independent extrafollicular response [148,180]. In a study by Caielli et al. (2018), it was found that the levels of IgG, IgA, and ABCs in the blood of SLE patients correlated with an increase in CD4 Th10 cells [109]. CD4 Th10 cells have been shown to be equally effective in inducing differentiation in B cells as Tfh cells [109] and have been identified in response to COVID-19 vaccines [184]. These cells induce differentiation of naïve and memory B cells into plasma cells with the help of IL-10 and succinate. Another study using human blood samples showed that a higher percentage of B cells from SLE patients expressed cell surface PLD4 compared with B cells from healthy donors [185]. Interestingly, these PLD4^+^ B cells largely overlapped with the ABC population, and it was found that stimulation of TLR7 or TLR9 could upregulate cell surface PLD4, indicating that PLD4^+^ B cells are probably TLR-stimulated autoreactive cells.

### 3.7. Targeting TLRs in the Treatment of SLE

Given the central role of TLR signaling in SLE disease, the past few decades have seen great interest in the development of drugs targeting the different TLRs [186,187]. Intriguingly, SLE drugs that are currently in clinical use in humans may also partly exert their effects by affecting TLR signaling. For instance, hydroxychloroquine, whose effect in cells is to increase the pH in acidic organelles, has been shown to prevent endosomal cleavage of TLR7, which in turn inhibits its function [188]. TLR9 is also cleaved in lysosomes and may likewise be inhibited by chloroquine [189]. Hydroxychloroquine has also been postulated to be able to inhibit presentation of autoantigens on MHC class II molecules through interfering with the formation of autoantigens in lysosomes [190]. In contrast, TLR signaling has been shown to dampen the effects of glucocorticoids, which are also commonly used to treat SLE patients [191]. Dual treatment with glucocorticoids and TLR antagonists may therefore be a promising strategy. Indeed, a recent preclinical study demonstrated that TLR7/8 inhibition increased the effect of glucocorticoids in lupus-prone mice and sensitized human PBMC against glucocorticoid treatment [192].

TLR antagonists also show potential as single agents. The targeting of TLR7/8 seems to be especially effective, and inhibitors of TLR7/8 are currently being tested in humans (NCT05638802, NCT05278663) [193]. Several other TLR-modulating drug candidates are also currently being developed and tested in murine models of SLE [194,195,196]. For instance, anti-TLR7 and anti-TLR9 antibodies have been tested in the NZBW model, where it was demonstrated that targeting TLR7 protected against LN while targeting of TLR9 had no effects [197]. In addition, a peptide derived from the core β sheet from TIRAP and conjugated to penetratin (a cell-penetrating peptide) was shown to block TLR4 signaling and subsequent cytokine response via inhibiting the MyD88 and TRIF-dependent pathways [198]. Clinical data from human trials with TLR antagonists are still relatively scarce, meaning that available reports on side effects are also limited. However, existing data imply that TLR antagonists are well tolerated and cause only mild side effects [193]. Inhibiting TLR activity has immunosuppressive effects. Thus, TLR antagonists may cause similar side effects to those of other immunosuppressive drugs, such as causing users to be more prone to infectious diseases and cancer [199]. Indeed, TLR agonists, including imiquimod, are used in cancer treatment [200], suggesting that TLR antagonism could instead have tumor-promoting effects. Hydroxychloroquine is considered safe and does not abrogate TLR signaling completely [190]. However, it can cause gastrointestinal problems like vomiting and diarrhea [201].

In addition, studies targeting pathways related to TLRs have been performed. For example, silencing HMGB1 expression in ovalbumin-induced asthmatic mice decreased expression of IgE and inflammatory factors [202]. In a review by Xue et al. (2021), different isoforms of HMGB1 are described with distinct physiological functions when released into the extracellular matrix, making it challenging to therapeutically target this protein [203]. The association between ABCs and autoantibody production in SLE has made these cells an interesting target for treatment of SLE. However, the markers currently used to identify this cell type are also shared by other immune cells and are therefore not specific enough to be used for targeting only ABCs [182]. Future work should therefore aim to identify ways of specifically targeting both ABCs and TLRs.

## 4. Conclusions and Future Directions

Due to its heterogenous nature, SLE is difficult to diagnose and treat efficiently, and animal models are invaluable for studying disease mechanisms and for testing novel therapeutics. Here, we have described various SLE mouse models, including spontaneous, genetically modified, inducible, and humanized models. Such models have contributed greatly to our knowledge about SLE and there are pronounced advantages to using mouse models when studying complex diseases.

The genetic and biological similarities between mice and humans are high as the genome of mice is 99% similar to the human genome, and the immune, endocrine, nervous, cardiovascular, and skeletal systems share similar complexity to the human systems. The reproducibility of mice and ease of breeding them, together with the use of modern sequencing and genomic engineering technologies to generate genetic alterations, allows us to utilize mouse models to research specific genetic targets of disease. However, mouse models do have limitations when compared with human SLE. Even though humans and mice are quite similar, we do not share the same immune system, and it is thus not possible to directly transfer results from mice to humans. The same goes for tolerance and response to treatment.

Current treatment of SLE often involves anti-inflammatory and immunosuppressive drugs, making the patients susceptible to infections. Environmental factors such as viruses and chemical elements resembling TLR ligands that can bind to and activate TLR and downstream signaling pathways may all contribute to SLE disease initiation and progression. As shown in an imiquimod-induced setting, both RNA and DNA sensing pathways may be activated in a TLR7-induced way without viral infection, indicating that other environmental stimuli such as chemicals can induce activation of cellular pathways involved in SLE. Evolutionarily developed bacterial and viral immune evasion strategies targeting TLR pathways may identify new compounds that can be used to stop or dampen any signaling aggravating the autoimmune disease.

The expression and interaction between the different TLRs, especially TLR7, TLR8, and TLR9, have a role in disease development in SLE. TLR7 acts as a disease-promoting factor, while TLR8 and TLR9 might have more regulating functions. Understanding these dynamics offers potential therapeutic targets for modulating immune responses in SLE and other autoimmune diseases. In addition, the dynamics between TLRs and other immune cells such as ABCs are important for disease etiology. Challenges remain in identifying the most appropriate targets for diagnosis, disease monitoring, and treatment. The fact that some of the TLRs have a tolerogenic function, such as TLR9, which may be responsible for the establishment of central B-cell tolerance, makes it an important target to restore B-cell tolerance in SLE and other autoimmune diseases. Thus, it is possible that dual inhibitors of TLR7 and TLR8 or TLR9 may be less effective than those that target only TLR7. It is therefore highly important to acquire more knowledge about the specific functions of individual TLRs.

In summary, understanding the interplay between environmental factors and pathway signaling and their involvement in SLE is necessary to provide insights into the mechanisms underlying disease flares and progression, potentially leading to the development of targeted therapies. Future studies using new technology and humanized mouse models have the potential to increase our knowledge of complex diseases such as SLE. However, while mouse models offer valuable insights and facilitate the exploration of specific genetic and molecular aspects of SLE, careful selection and interpretation of these models are crucial for advancing our understanding and treatment of SLE, exemplified by the different results regarding autoantibody repertoire in different SLE mouse models, both before and after knockout of TLRs.

## References

[1] Aringer M., Costenbader K., Daikh D., Brinks R., Mosca M., Ramsey-Goldman R., Smolen J.S., Wofsy D., Boumpas D.T., Kamen D.L. (2019). 2019 European League Against Rheumatism/American College of Rheumatology classification criteria for systemic lupus erythematosus. Ann. Rheum. Dis..

[2] Dema B., Charles N. (2016). Autoantibodies in SLE: Specificities, Isotypes and Receptors. Antibodies.

[3] Nowling T.K., Gilkeson G.S. (2011). Mechanisms of tissue injury in lupus nephritis. Arthritis Res. Ther..

[4] Duan T., Du Y., Xing C., Wang H.Y., Wang R.-F. (2022). Toll-Like Receptor Signaling and Its Role in Cell-Mediated Immunity. Front. Immunol..

[5] Kawai T., Ikegawa M., Ori D., Akira S. (2024). Decoding Toll-like receptors: Recent insights and perspectives in innate immunity. Immunity.

[6] Su Y.J., Li F.A., Sheu J.J., Li S.C., Weng S.W., Shen F.C., Chang Y.H., Chen H.Y., Liou C.W., Lin T.K. (2022). A Study on MDA5 Signaling in Splenic B Cells from an Imiquimod-Induced Lupus Mouse Model with Proteomics. Cells.

[7] Moore E., Putterman C. (2020). Are lupus animal models useful for understanding and developing new therapies for human SLE?. J. Autoimmun..

[8] Du Y., Sanam S., Kate K., Mohan C. (2015). Animal models of lupus and lupus nephritis. Curr. Pharm. Des..

[9] Li W., Titov A.A., Morel L. (2017). An update on lupus animal models. Curr. Opin. Rheumatol..

[10] Richard M.L., Gilkeson G. (2018). Mouse models of lupus: What they tell us and what they don’t. Lupus Sci. Med..

[11] Halkom A., Wu H., Lu Q. (2020). Contribution of mouse models in our understanding of lupus. Int. Rev. Immunol..

[12] Katikaneni D., Morel L., Scindia Y. (2024). Animal models of lupus nephritis: The past, present and a future outlook. Autoimmunity.

[13] Helyer B.J., Howie J.B. (1963). Renal disease associated with positive lupus erythematosus tests in a cross-bred strain of mice. Nature.

[14] Dubois E.L., Horowitz R.E., Demopoulos H.B., Teplitz R. (1966). NZB/NZW mice as a model of systemic lupus erythematosus. JAMA.

[15] Morel L. (2012). Mapping lupus susceptibility genes in the NZM2410 mouse model. Adv. Immunol..

[16] Vidal S., Gelpi C., Rodriguez-Sanchez J.L. (1994). (SWR × SJL)F1 mice: A new model of lupus-like disease. J. Exp. Med..

[17] Andrews B.S., Eisenberg R.A., Theofilopoulos A.N., Izui S., Wilson C.B., McConahey P.J., Murphy E.D., Roths J.B., Dixon F.J. (1978). Spontaneous murine lupus-like syndromes. Clinical and immunopathological manifestations in several strains. J. Exp. Med..

[18] Bolland S., Yim Y.S., Tus K., Wakeland E.K., Ravetch J.V. (2002). Genetic modifiers of systemic lupus erythematosus in FcgammaRIIB(-/-) mice. J. Exp. Med..

[19] Ondee T., Surawut S., Taratummarat S., Hirankarn N., Palaga T., Pisitkun P., Pisitkun T., Leelahavanichkul A. (2017). Fc Gamma Receptor IIB Deficient Mice: A Lupus Model with Increased Endotoxin Tolerance-Related Sepsis Susceptibility. Shock.

[20] Han J.H., Umiker B.R., Kazimirova A.A., Fray M., Korgaonkar P., Selsing E., Imanishi-Kari T. (2014). Expression of an anti-RNA autoantibody in a mouse model of SLE increases neutrophil and monocyte numbers as well as IFN-I expression. Eur. J. Immunol..

[21] Deane J.A., Pisitkun P., Barrett R.S., Feigenbaum L., Town T., Ward J.M., Flavell R.A., Bolland S. (2007). Control of Toll-like Receptor 7 Expression Is Essential to Restrict Autoimmunity and Dendritic Cell Proliferation. Immunity.

[22] Tizaoui K., Terrazzino S., Cargnin S., Lee K.H., Gauckler P., Li H., Shin J.I., Kronbichler A. (2021). The role of PTPN22 in the pathogenesis of autoimmune diseases: A comprehensive review. Semin. Arthritis Rheum..

[23] Wang Y., Shaked I., Stanford S.M., Zhou W., Curtsinger J.M., Mikulski Z., Shaheen Z.R., Cheng G., Sawatzke K., Campbell A.M. (2013). The autoimmunity-associated gene PTPN22 potentiates toll-like receptor-driven, type 1 interferon-dependent immunity. Immunity.

[24] Zheng J., Petersen F., Yu X. (2014). The role of PTPN22 in autoimmunity: Learning from mice. Autoimmun. Rev..

[25] Wilber A., O’Connor T.P., Lu M.L., Karimi A., Schneider M.C. (2003). Dnase1l3 deficiency in lupus-prone MRL and NZB/W F1 mice. Clin. Exp. Immunol..

[26] Engavale M., Hernandez C.J., Infante A., LeRoith T., Radovan E., Evans L., Villarreal J., Reilly C.M., Sutton R.B., Keyel P.A. (2023). Deficiency of macrophage-derived Dnase1L3 causes lupus-like phenotypes in mice. J. Leukoc. Biol..

[27] Sisirak V., Sally B., D’Agati V., Martinez-Ortiz W., Ozcakar Z.B., David J., Rashidfarrokhi A., Yeste A., Panea C., Chida A.S. (2016). Digestion of Chromatin in Apoptotic Cell Microparticles Prevents Autoimmunity. Cell.

[28] Weisenburger T., von Neubeck B., Schneider A., Ebert N., Schreyer D., Acs A., Winkler T.H. (2018). Epistatic Interactions Between Mutations of Deoxyribonuclease 1-Like 3 and the Inhibitory Fc Gamma Receptor IIB Result in Very Early and Massive Autoantibodies Against Double-Stranded DNA. Front. Immunol..

[29] Korn M.A., Steffensen M., Brandl C., Royzman D., Daniel C., Winkler T.H., Nitschke L. (2023). Epistatic effects of Siglec-G and DNase1 or DNase1l3 deficiencies in the development of systemic lupus erythematosus. Front. Immunol..

[30] Napirei M., Karsunky H., Zevnik B., Stephan H., Mannherz H.G., Moroy T. (2000). Features of systemic lupus erythematosus in Dnase1-deficient mice. Nat. Genet..

[31] Kenny E.F., Raupach B., Abu Abed U., Brinkmann V., Zychlinsky A. (2019). Dnase1-deficient mice spontaneously develop a systemic lupus erythematosus-like disease. Eur. J. Immunol..

[32] Hibbs M.L., Tarlinton D.M., Armes J., Grail D., Hodgson G., Maglitto R., Stacker S.A., Dunn A.R. (1995). Multiple defects in the immune system of Lyn-deficient mice, culminating in autoimmune disease. Cell.

[33] Ban T., Sato G.R., Nishiyama A., Akiyama A., Takasuna M., Umehara M., Suzuki S., Ichino M., Matsunaga S., Kimura A. (2016). Lyn Kinase Suppresses the Transcriptional Activity of IRF5 in the TLR-MyD88 Pathway to Restrain the Development of Autoimmunity. Immunity.

[34] Park J.S., Yang S., Hwang S.H., Choi J., Kwok S.K., Kong Y.Y., Youn J., Cho M.L., Park S.H. (2022). B Cell-Specific Deletion of CR6-Interacting Factor 1 Drives Lupus-like Autoimmunity by Activation of Interleukin-17, Interleukin-6, and Pathogenic Follicular Helper T Cells in a Mouse Model. Arthritis Rheumatol..

[35] Lietke D.S. (2017). CRIF1 and Its Function in Anti-Viral Immunity.

[36] Lee K., Park J., Tanno H., Georgiou G., Diamond B., Kim S.J. (2023). Peripheral T cell activation, not thymic selection, expands the T follicular helper repertoire in a lupus-prone murine model. Proc. Natl. Acad. Sci. USA.

[37] Kim S.J., Schatzle S., Ahmed S.S., Haap W., Jang S.H., Gregersen P.K., Georgiou G., Diamond B. (2017). Increased cathepsin S in Prdm1(-/-) dendritic cells alters the T(FH) cell repertoire and contributes to lupus. Nat. Immunol..

[38] Kim V., Lee K., Tian H., Jang S.H., Diamond B., Kim S.J. (2022). IL-17-producing follicular Th cells enhance plasma cell differentiation in lupus-prone mice. JCI Insight.

[39] Ko Y.A., Chan Y.H., Liu C.H., Liang J.J., Chuang T.H., Hsueh Y.P., Lin Y.L., Lin K.I. (2018). Blimp-1-Mediated Pathway Promotes Type I IFN Production in Plasmacytoid Dendritic Cells by Targeting to Interleukin-1 Receptor-Associated Kinase M. Front. Immunol..

[40] Reuschle Q., Van Heddegem L., Bosteels V., Moncan M., Depauw S., Wadier N., Marechal S., De Nolf C., Delgado V., Messai Y. (2024). Loss of function of XBP1 splicing activity of IRE1alpha favors B cell tolerance breakdown. J. Autoimmun..

[41] Murga M., Fernandez-Capetillo O., Field S.J., Moreno B., Borlado L.R., Fujiwara Y., Balomenos D., Vicario A., Carrera A.C., Orkin S.H. (2001). Mutation of E2F2 in mice causes enhanced T lymphocyte proliferation, leading to the development of autoimmunity. Immunity.

[42] Marin-Vidalled M.J., Bolivar A., Zubiaga A., Lopez-Hoyos M. (2010). The combined effect of BCL-2 over-expression and E2F2 deficiency induces an autoimmune syndrome in non-susceptible mouse strain C57BL/6. Autoimmunity.

[43] Wang S., Wang L., Wu C., Sun S., Pan J.H. (2018). E2F2 directly regulates the STAT1 and PI3K/AKT/NF-kappaB pathways to exacerbate the inflammatory phenotype in rheumatoid arthritis synovial fibroblasts and mouse embryonic fibroblasts. Arthritis Res. Ther..

[44] Akizuki S., Ishigaki K., Kochi Y., Law S.M., Matsuo K., Ohmura K., Suzuki A., Nakayama M., Iizuka Y., Koseki H. (2019). PLD4 is a genetic determinant to systemic lupus erythematosus and involved in murine autoimmune phenotypes. Ann. Rheum. Dis..

[45] Gavin A.L., Blane T.R., Thinnes T.C., Gerlt E., Marshak-Rothstein A., Huang D., Nemazee D. (2023). Disease in the Pld4thss/thss Model of Murine Lupus Requires TLR9. Immunohorizons.

[46] Gavin A.L., Huang D., Huber C., Martensson A., Tardif V., Skog P.D., Blane T.R., Thinnes T.C., Osborn K., Chong H.S. (2018). PLD3 and PLD4 are single-stranded acid exonucleases that regulate endosomal nucleic-acid sensing. Nat. Immunol..

[47] Yu P., Constien R., Dear N., Katan M., Hanke P., Bunney T.D., Kunder S., Quintanilla-Martinez L., Huffstadt U., Schroder A. (2005). Autoimmunity and inflammation due to a gain-of-function mutation in phospholipase C gamma 2 that specifically increases external Ca2+ entry. Immunity.

[48] Becker-Herman S., Meyer-Bahlburg A., Schwartz M.A., Jackson S.W., Hudkins K.L., Liu C., Sather B.D., Khim S., Liggitt D., Song W. (2011). WASp-deficient B cells play a critical, cell-intrinsic role in triggering autoimmunity. J. Exp. Med..

[49] Jackson S.W., Scharping N.E., Kolhatkar N.S., Khim S., Schwartz M.A., Li Q.-Z., Hudkins K.L., Alpers C.E., Liggitt D., Rawlings D.J. (2014). Opposing Impact of B Cell–Intrinsic TLR7 and TLR9 Signals on Autoantibody Repertoire and Systemic Inflammation. J. Immunol..

[50] Jiang Y., Xiang Y., Lin C., Zhang W., Yang Z., Xiang L., Xiao Y., Chen L., Ran Q., Li Z. (2022). Multifunctions of CRIF1 in cancers and mitochondrial dysfunction. Front. Oncol..

[51] Nadeau S., Martins G.A. (2021). Conserved and Unique Functions of Blimp1 in Immune Cells. Front. Immunol..

[52] Brodie E.J., Infantino S., Low M.S.Y., Tarlinton D.M. (2018). Lyn, Lupus, and (B) Lymphocytes, a Lesson on the Critical Balance of Kinase Signaling in Immunity. Front. Immunol..

[53] Jackson J.T., Mulazzani E., Nutt S.L., Masters S.L. (2021). The role of PLCgamma2 in immunological disorders, cancer, and neurodegeneration. J. Biol. Chem..

[54] Rey-Suarez I., Wheatley B.A., Koo P., Bhanja A., Shu Z., Mochrie S., Song W., Shroff H., Upadhyaya A. (2020). WASP family proteins regulate the mobility of the B cell receptor during signaling activation. Nat. Commun..

[55] Junjappa R.P., Patil P., Bhattarai K.R., Kim H.R., Chae H.J. (2018). IRE1alpha Implications in Endoplasmic Reticulum Stress-Mediated Development and Pathogenesis of Autoimmune Diseases. Front. Immunol..

[56] Park S.M., Kang T.I., So J.S. (2021). Roles of XBP1s in Transcriptional Regulation of Target Genes. Biomedicines.

[57] Freitas E.C., de Oliveira M.S., Monticielo O.A. (2017). Pristane-induced lupus: Considerations on this experimental model. Clin. Rheumatol..

[58] Satoh M., Richards H.B., Shaheen V.M., Yoshida H., Shaw M., Naim J.O., Wooley P.H., Reeves W.H. (2000). Widespread susceptibility among inbred mouse strains to the induction of lupus autoantibodies by pristane. Clin. Exp. Immunol..

[59] Bender A.T., Wu Y., Cao Q., Ding Y., Oestreicher J., Genest M., Akare S., Ishizaka S.T., Mackey M.F. (2014). Assessment of the translational value of mouse lupus models using clinically relevant biomarkers. Transl. Res..

[60] Postal M., Vivaldo J.F., Fernandez-Ruiz R., Paredes J.L., Appenzeller S., Niewold T.B. (2020). Type I interferon in the pathogenesis of systemic lupus erythematosus. Curr. Opin. Immunol..

[61] Savarese E., Steinberg C., Pawar R.D., Reindl W., Akira S., Anders H.J., Krug A. (2008). Requirement of Toll-like receptor 7 for pristane-induced production of autoantibodies and development of murine lupus nephritis. Arthritis Rheum..

[62] Urbonaviciute V., Starke C., Pirschel W., Pohle S., Frey S., Daniel C., Amann K., Schett G., Herrmann M., Voll R.E. (2013). Toll-like receptor 2 is required for autoantibody production and development of renal disease in pristane-induced lupus. Arthritis Rheum..

[63] Summers S.A., Hoi A., Steinmetz O.M., O’Sullivan K.M., Ooi J.D., Odobasic D., Akira S., Kitching A.R., Holdsworth S.R. (2010). TLR9 and TLR4 are required for the development of autoimmunity and lupus nephritis in pristane nephropathy. J. Autoimmun..

[64] Bossaller L., Christ A., Pelka K., Nündel K., Chiang P.I., Pang C., Mishra N., Busto P., Bonegio R.G., Schmidt R.E. (2016). TLR9 Deficiency Leads to Accelerated Renal Disease and Myeloid Lineage Abnormalities in Pristane-Induced Murine Lupus. J. Immunol..

[65] Giordano D., Kuley R., Draves K.E., Elkon K.B., Giltiay N.V., Clark E.A. (2023). B cell-activating factor (BAFF) from dendritic cells, monocytes and neutrophils is required for B cell maturation and autoantibody production in SLE-like autoimmune disease. Front. Immunol..

[66] Yoo E.J., Oh K.H., Piao H., Kang H.J., Jeong G.W., Park H., Lee C.J., Ryu H., Yang S.H., Kim M.G. (2023). Macrophage transcription factor TonEBP promotes systemic lupus erythematosus and kidney injury via damage-induced signaling pathways. Kidney Int..

[67] Miyagawa F., Tagaya Y., Ozato K., Asada H. (2016). Essential Requirement for IFN Regulatory Factor 7 in Autoantibody Production but Not Development of Nephritis in Murine Lupus. J. Immunol..

[68] Motwani M., McGowan J., Antonovitch J., Gao K.M., Jiang Z., Sharma S., Baltus G.A., Nickerson K.M., Marshak-Rothstein A., Fitzgerald K.A. (2021). cGAS-STING Pathway Does Not Promote Autoimmunity in Murine Models of SLE. Front. Immunol..

[69] Corzo C.A., Varfolomeev E., Setiadi A.F., Francis R., Klabunde S., Senger K., Sujatha-Bhaskar S., Drobnick J., Do S., Suto E. (2020). The kinase IRAK4 promotes endosomal TLR and immune complex signaling in B cells and plasmacytoid dendritic cells. Sci. Signal.

[70] Lin W., Seshasayee D., Lee W.P., Caplazi P., McVay S., Suto E., Nguyen A., Lin Z., Sun Y., DeForge L. (2015). Dual B cell immunotherapy is superior to individual anti-CD20 depletion or BAFF blockade in murine models of spontaneous or accelerated lupus. Arthritis Rheumatol..

[71] Gardet A., Chou W.C., Reynolds T.L., Velez D.B., Fu K., Czerkowicz J.M., Bajko J., Ranger A.M., Allaire N., Kerns H.M. (2016). Pristane-Accelerated Autoimmune Disease in (SWR X NZB) F1 Mice Leads to Prominent Tubulointerstitial Inflammation and Human Lupus Nephritis-Like Fibrosis. PLoS ONE.

[72] Satoh M., Weintraub J.P., Yoshida H., Shaheen V.M., Richards H.B., Shaw M., Reeves W.H. (2000). Fas and Fas ligand mutations inhibit autoantibody production in pristane-induced lupus. J. Immunol..

[73] Yokogawa M., Takaishi M., Nakajima K., Kamijima R., Fujimoto C., Kataoka S., Terada Y., Sano S. (2014). Epicutaneous application of toll-like receptor 7 agonists leads to systemic autoimmunity in wild-type mice: A new model of systemic Lupus erythematosus. Arthritis Rheumatol..

[74] Gleichmann E., Gleichmann H. (1985). Pathogenesis of graft-versus-host reactions (GVHR) and GVH-like diseases. J. Invest. Dermatol..

[75] Srinivasan M., Flynn R., Price A., Ranger A., Browning J.L., Taylor P.A., Ritz J., Antin J.H., Murphy W.J., Luznik L. (2012). Donor B-cell alloantibody deposition and germinal center formation are required for the development of murine chronic GVHD and bronchiolitis obliterans. Blood.

[76] Bracken S.J., Suthers A.N., DiCioccio R.A., Su H., Anand S., Poe J.C., Jia W., Visentin J., Basher F., Jordan C.Z. (2024). Heightened TLR7 signaling primes BCR-activated B cells in chronic graft-versus-host disease for effector functions. Blood Adv..

[77] Garimella M.G., He C., Chen G., Li Q.Z., Huang X., Karlsson M.C.I. (2021). The B cell response to both protein and nucleic acid antigens displayed on apoptotic cells are dependent on endosomal pattern recognition receptors. J. Autoimmun..

[78] Hayakawa K., Fujishiro M., Yoshida Y., Kataoka Y., Sakuma S., Nishi T., Ikeda K., Morimoto S., Takamori K., Sekigawa I. (2022). Exposure of female NZBWF1 mice to imiquimod-induced lupus nephritis at an early age via a unique mechanism that differed from spontaneous onset. Clin. Exp. Immunol..

[79] Ma L., Terrell M., Brown J., Castellanos Garcia A., Elshikha A., Morel L. (2023). TLR7/TLR8 activation and susceptibility genes synergize to breach gut barrier in a mouse model of lupus. Front. Immunol..

[80] Liu Z., Bethunaickan R., Huang W., Ramanujam M., Madaio M.P., Davidson A. (2011). IFN-alpha confers resistance of systemic lupus erythematosus nephritis to therapy in NZB/W F1 mice. J. Immunol..

[81] Pollard K.M., Escalante G.M., Huang H., Haraldsson K.M., Hultman P., Christy J.M., Pawar R.D., Mayeux J.M., Gonzalez-Quintial R., Baccala R. (2017). Induction of Systemic Autoimmunity by a Xenobiotic Requires Endosomal TLR Trafficking and Signaling from the Late Endosome and Endolysosome but Not Type I IFN. J. Immunol..

[82] Gill R.F., Mathieu P.A., Lash L.H., Rosenspire A.J. (2023). Naturally occurring autoimmune disease in (NZB X NZW) F1 mice is correlated with suppression of MZ B cell development due to aberrant B Cell Receptor (BCR) signaling, which is exacerbated by exposure to inorganic mercury. Toxicol. Sci..

[83] Favor O.K., Chauhan P.S., Pourmand E., Edwards A.M., Wagner J.G., Lewandowski R.P., Heine L.K., Harkema J.R., Lee K.S.S., Pestka J.J. (2023). Lipidome modulation by dietary omega-3 polyunsaturated fatty acid supplementation or selective soluble epoxide hydrolase inhibition suppresses rough LPS-accelerated glomerulonephritis in lupus-prone mice. Front. Immunol..

[84] Hasegawa K., Hayashi T. (2003). Synthetic CpG oligodeoxynucleotides accelerate the development of lupus nephritis during preactive phase in NZB x NZWF1 mice. Lupus.

[85] Chen J., Liao S., Zhou H., Yang L., Guo F., Chen S., Li A., Pan Q., Yang C., Liu H.F. (2021). Humanized Mouse Models of Systemic Lupus Erythematosus: Opportunities and Challenges. Front. Immunol..

[86] Maria N.I., Papoin J., Raparia C., Sun Z., Josselsohn R., Lu A., Katerji H., Syeda M.M., Polsky D., Paulson R. (2023). Human TLR8 induces inflammatory bone marrow erythromyeloblastic islands and anemia in SLE-prone mice. Life Sci. Alliance.

[87] Cakan E., Ah Kioon M.D., Garcia-Carmona Y., Glauzy S., Oliver D., Yamakawa N., Vega Loza A., Du Y., Schickel J.N., Boeckers J.M. (2023). TLR9 ligand sequestration by chemokine CXCL4 negatively affects central B cell tolerance. J. Exp. Med..

[88] Schaper F., de Leeuw K., Horst G., Maas F., Bootsma H., Heeringa P., Limburg P.C., Westra J. (2017). Autoantibodies to box A of high mobility group box 1 in systemic lupus erythematosus. Clin. Exp. Immunol..

[89] Abdulahad D.A., Westra J., Bijzet J., Limburg P.C., Kallenberg C.G., Bijl M. (2011). High mobility group box 1 (HMGB1) and anti-HMGB1 antibodies and their relation to disease characteristics in systemic lupus erythematosus. Arthritis Res. Ther..

[90] Wirestam L., Schierbeck H., Skogh T., Gunnarsson I., Ottosson L., Erlandsson-Harris H., Wettero J., Sjowall C. (2015). Antibodies against High Mobility Group Box protein-1 (HMGB1) versus other anti-nuclear antibody fine-specificities and disease activity in systemic lupus erythematosus. Arthritis Res. Ther..

[91] Tanaka A., Ito T., Kibata K., Inagaki-Katashiba N., Amuro H., Nishizawa T., Son Y., Ozaki Y., Nomura S. (2019). Serum high-mobility group box 1 is correlated with interferon-alpha and may predict disease activity in patients with systemic lupus erythematosus. Lupus.

[92] Urbonaviciute V., Furnrohr B.G., Meister S., Munoz L., Heyder P., De Marchis F., Bianchi M.E., Kirschning C., Wagner H., Manfredi A.A. (2008). Induction of inflammatory and immune responses by HMGB1-nucleosome complexes: Implications for the pathogenesis of SLE. J. Exp. Med..

[93] Das N., Dewan V., Grace P.M., Gunn R.J., Tamura R., Tzarum N., Watkins L.R., Wilson I.A., Yin H. (2016). HMGB1 Activates Proinflammatory Signaling via TLR5 Leading to Allodynia. Cell Rep..

[94] Ma K., Li J., Wang X., Lin X., Du W., Yang X., Mou F., Fang Y., Zhao Y., Hong X. (2018). TLR4(+)CXCR4(+) plasma cells drive nephritis development in systemic lupus erythematosus. Ann. Rheum. Dis..

[95] Li S.J., Ruan D.D., Wu W.Z., Wu M., Wu Q.Y., Wang H.L., Ji Y.Y., Zhang Y.P., Lin X.F., Fang Z.T. (2023). Potential regulatory role of the Nrf2/HMGB1/TLR4/NF-kappaB signaling pathway in lupus nephritis. Pediatr. Rheumatol. Online J..

[96] Huang Q., Shen S., Qu H., Huang Y., Wu D., Jiang H., Yuan C. (2020). Expression of HMGB1 and TLR4 in neuropsychiatric systemic lupus erythematosus patients with seizure disorders. Ann. Transl. Med..

[97] Elloumi N., Tahri S., Fakhfakh R., Abida O., Mahfoudh N., Hachicha H., Marzouk S., Bahloul Z., Masmoudi H. (2022). Role of innate immune receptors TLR4 and TLR2 polymorphisms in systemic lupus erythematosus susceptibility. Ann. Hum. Genet..

[98] Lee Y.H., Lee H.S., Choi S.J., Ji J.D., Song G.G. (2012). Associations between TLR polymorphisms and systemic lupus erythematosus: A systematic review and meta-analysis. Clin. Exp. Rheumatol..

[99] Sanchez E., Orozco G., Lopez-Nevot M.A., Jimenez-Alonso J., Martin J. (2004). Polymorphisms of toll-like receptor 2 and 4 genes in rheumatoid arthritis and systemic lupus erythematosus. Tissue Antigens.

[100] Alajoleen R.M., Oakland D.N., Estaleen R., Shakeri A., Lu R., Appiah M., Sun S., Neumann J., Kawauchi S., Cecere T.E. (2024). Tlr5 deficiency exacerbates lupus-like disease in the MRL/lpr mouse model. Front. Immunol..

[101] Patole P.S., Pawar R.D., Lech M., Zecher D., Schmidt H., Segerer S., Ellwart A., Henger A., Kretzler M., Anders H.J. (2006). Expression and regulation of Toll-like receptors in lupus-like immune complex glomerulonephritis of MRL-Fas(lpr) mice. Nephrol. Dial. Transpl..

[102] Elloumi N., Fakhfakh R., Abida O., Ayadi L., Marzouk S., Hachicha H., Fourati M., Bahloul Z., Mhiri M.N., Kammoun K. (2017). Relevant genetic polymorphisms and kidney expression of Toll-like receptor (TLR)-5 and TLR-9 in lupus nephritis. Clin. Exp. Immunol..

[103] Rupasree Y., Naushad S.M., Varshaa R., Mahalakshmi G.S., Kumaraswami K., Rajasekhar L., Kutala V.K. (2016). Application of Various Statistical Models to Explore Gene-Gene Interactions in Folate, Xenobiotic, Toll-Like Receptor and STAT4 Pathways that Modulate Susceptibility to Systemic Lupus Erythematosus. Mol. Diagn. Ther..

[104] Hou Y., Wang L., Luo C., Tang W., Dai R., An Y., Tang X. (2023). Clinical characteristics of early-onset paediatric systemic lupus erythematosus in a single centre in China. Rheumatology.

[105] Oldenburg M., Kruger A., Ferstl R., Kaufmann A., Nees G., Sigmund A., Bathke B., Lauterbach H., Suter M., Dreher S. (2012). TLR13 recognizes bacterial 23S rRNA devoid of erythromycin resistance-forming modification. Science.

[106] Li X.D., Chen Z.J. (2012). Sequence specific detection of bacterial 23S ribosomal RNA by TLR13. Elife.

[107] Lee S.M., Yip T.F., Yan S., Jin D.Y., Wei H.L., Guo R.T., Peiris J.S.M. (2018). Recognition of Double-Stranded RNA and Regulation of Interferon Pathway by Toll-Like Receptor 10. Front. Immunol..

[108] Rodrigues C.R., Balachandran Y., Aulakh G.K., Singh B. (2024). TLR10: An intriguing Toll-like receptor with many unanswered questions. J. Innate Immun..

[109] Caielli S., Wan Z., Pascual V. (2023). Systemic Lupus Erythematosus Pathogenesis: Interferon and Beyond. Annu. Rev. Immunol..

[110] Scherlinger M., Guillotin V., Truchetet M.E., Contin-Bordes C., Sisirak V., Duffau P., Lazaro E., Richez C., Blanco P. (2018). Systemic lupus erythematosus and systemic sclerosis: All roads lead to platelets. Autoimmun. Rev..

[111] Robert M., Scherlinger M. (2024). Platelets are a major player and represent a therapeutic opportunity in systemic lupus erythematosus. Jt. Bone Spine.

[112] Linge P., Fortin P.R., Lood C., Bengtsson A.A., Boilard E. (2018). The non-haemostatic role of platelets in systemic lupus erythematosus. Nat. Rev. Rheumatol..

[113] Scherlinger M., Richez C., Tsokos G.C., Boilard E., Blanco P. (2023). The role of platelets in immune-mediated inflammatory diseases. Nat. Rev. Immunol..

[114] Baroni Pietto M.C., Glembotsky A.C., Lev P.R., Marin Oyarzun C.R., De Luca G., Gomez G., Collado M.V., Charo N., Cellucci A.S., Heller P.G. (2024). Toll-like receptor expression and functional behavior in platelets from patients with systemic lupus erythematosus. Immunobiology.

[115] Hornung V., Rothenfusser S., Britsch S., Krug A., Jahrsdöfer B., Giese T., Endres S., Hartmann G. (2002). Quantitative Expression of Toll-Like Receptor 1–10 mRNA in Cellular Subsets of Human Peripheral Blood Mononuclear Cells and Sensitivity to CpG Oligodeoxynucleotides1. J. Immunol..

[116] Demaria O., Pagni P.P., Traub S., de Gassart A., Branzk N., Murphy A.J., Valenzuela D.M., Yancopoulos G.D., Flavell R.A., Alexopoulou L. (2010). TLR8 deficiency leads to autoimmunity in mice. J. Clin. Investig..

[117] Tilstra J.S., John S., Gordon R.A., Leibler C., Kashgarian M., Bastacky S., Nickerson K.M., Shlomchik M.J. (2020). B cell-intrinsic TLR9 expression is protective in murine lupus. J. Clin. Investig..

[118] Heil F., Hemmi H., Hochrein H., Ampenberger F., Kirschning C., Akira S., Lipford G., Wagner H., Bauer S. (2004). Species-Specific Recognition of Single-Stranded RNA via Toll-like Receptor 7 and 8. Science.

[119] Peng S.L. (2005). Signaling in B cells via Toll-like receptors. Curr. Opin. Immunol..

[120] Wen L., Zhang B., Wu X., Liu R., Fan H., Han L., Zhang Z., Ma X., Chu C.-Q., Shi X. (2023). Toll-like receptors 7 and 9 regulate the proliferation and differentiation of B cells in systemic lupus erythematosus. Front. Immunol..

[121] Rubin S.J.S., Bloom M.S., Robinson W.H. (2019). B cell checkpoints in autoimmune rheumatic diseases. Nat. Rev. Rheumatol..

[122] Leadbetter E.A., Rifkin I.R., Hohlbaum A.M., Beaudette B.C., Shlomchik M.J., Marshak-Rothstein A. (2002). Chromatin–IgG complexes activate B cells by dual engagement of IgM and Toll-like receptors. Nature.

[123] Viglianti G.A., Lau C.M., Hanley T.M., Miko B.A., Shlomchik M.J., Marshak-Rothstein A. (2003). Activation of Autoreactive B Cells by CpG dsDNA. Immunity.

[124] Christensen S.R., Shupe J., Nickerson K., Kashgarian M., Flavell R.A., Shlomchik M.J. (2006). Toll-like Receptor 7 and TLR9 Dictate Autoantibody Specificity and Have Opposing Inflammatory and Regulatory Roles in a Murine Model of Lupus. Immunity.

[125] Lau C.M., Broughton C., Tabor A.S., Akira S., Flavell R.A., Mamula M.J., Christensen S.R., Shlomchik M.J., Viglianti G.A., Rifkin I.R. (2005). RNA-associated autoantigens activate B cells by combined B cell antigen receptor/Toll-like receptor 7 engagement. J. Exp. Med..

[126] Lin L., Gerth A.J., Peng S.L. (2004). CpG DNA redirects class-switching towards ‘Th1-like’ Ig isotype production via TLR9 and MyD88. Eur. J. Immunol..

[127] He B., Qiao X., Cerutti A. (2004). CpG DNA Induces IgG Class Switch DNA Recombination by Activating Human B Cells through an Innate Pathway That Requires TLR9 and Cooperates with IL-101. J. Immunol..

[128] Ehlers M., Fukuyama H., McGaha T.L., Aderem A., Ravetch J.V. (2006). TLR9/MyD88 signaling is required for class switching to pathogenic IgG2a and 2b autoantibodies in SLE. J. Exp. Med..

[129] Eckl-Dorna J., Batista F.D. (2009). BCR-mediated uptake of antigen linked to TLR9 ligand stimulates B-cell proliferation and antigen-specific plasma cell formation. Blood.

[130] Rivera C.E., Zhou Y., Chupp D.P., Yan H., Fisher A.D., Simon R., Zan H., Xu Z., Casali P. (2023). Intrinsic B cell TLR-BCR linked coengagement induces class-switched, hypermutated, neutralizing antibody responses in absence of T cells. Sci. Adv..

[131] Christensen S.R., Kashgarian M., Alexopoulou L., Flavell R.A., Akira S., Shlomchik M.J. (2005). Toll-like receptor 9 controls anti-DNA autoantibody production in murine lupus. J. Exp. Med..

[132] Rahman A.H., Eisenberg R.A. (2006). The role of toll-like receptors in systemic lupus erythematosus. Springer Semin. Immunopathol..

[133] Yi A.-K., Chang M., Peckham D.W., Krieg A.M., Ashman R.F. (1998). CpG Oligodeoxyribonucleotides Rescue Mature Spleen B Cells from Spontaneous Apoptosis and Promote Cell Cycle Entry1. J. Immunol..

[134] Han S.-S., Chung S.-T., Robertson D.A., Chelvarajan R.L., Bondada S. (1999). CpG oligodeoxynucleotides rescue BKS-2 immature B cell lymphoma from anti-IgM-mediated growth inhibition by up-regulation of egr-1. Int. Immunol..

[135] Nickerson K.M., Christensen S.R., Shupe J., Kashgarian M., Kim D., Elkon K., Shlomchik M.J. (2010). TLR9 Regulates TLR7- and MyD88-Dependent Autoantibody Production and Disease in a Murine Model of Lupus. J. Immunol..

[136] Teichmann L.L., Schenten D., Medzhitov R., Kashgarian M., Shlomchik M.J. (2013). Signals via the adaptor MyD88 in B cells and DCs make distinct and synergistic contributions to immune activation and tissue damage in lupus. Immunity.

[137] Tilstra J.S., Kim M., Gordon R.A., Leibler C., Cosgrove H.A., Bastacky S., Nickerson K.M., Shlomchik M.J. (2023). B cell–intrinsic Myd88 regulates disease progression in murine lupus. J. Exp. Med..

[138] Hwang S.-H., Lee H., Yamamoto M., Jones L.A., Dayalan J., Hopkins R., Zhou X.J., Yarovinsky F., Connolly J.E., Curotto de Lafaille M.A. (2012). B Cell TLR7 Expression Drives Anti-RNA Autoantibody Production and Exacerbates Disease in Systemic Lupus Erythematosus–Prone Mice. J. Immunol..

[139] Satterthwaite A.B. (2021). TLR7 Signaling in Lupus B Cells: New Insights into Synergizing Factors and Downstream Signals. Curr. Rheumatol. Rep..

[140] Desnues B., Macedo A.B., Roussel-Queval A., Bonnardel J., Henri S., Demaria O., Alexopoulou L. (2014). TLR8 on dendritic cells and TLR9 on B cells restrain TLR7-mediated spontaneous autoimmunity in C57BL/6 mice. Proc. Natl. Acad. Sci. USA.

[141] Scofield R.H., Bruner G.R., Namjou B., Kimberly R.P., Ramsey-Goldman R., Petri M., Reveille J.D., Alarcón G.S., Vilá L.M., Reid J. (2008). Klinefelter’s syndrome (47,XXY) in male systemic lupus erythematosus patients: Support for the notion of a gene-dose effect from the X chromosome. Arthritis Rheum..

[142] Souyris M., Cenac C., Azar P., Daviaud D., Canivet A., Grunenwald S., Pienkowski C., Chaumeil J., Mejía J.E., Guéry J.-C. (2018). TLR7 escapes X chromosome inactivation in immune cells. Sci. Immunol..

[143] Youness A., Cenac C., Faz-López B., Grunenwald S., Barrat F.J., Chaumeil J., Mejía J.E., Guéry J.-C. (2023). TLR8 escapes X chromosome inactivation in human monocytes and CD4+ T cells. Biol. Sex. Differ..

[144] Wang T., Marken J., Chen J., Tran V.B., Li Q.-Z., Li M., Cerosaletti K., Elkon K.B., Zeng X., Giltiay N.V. (2019). High TLR7 Expression Drives the Expansion of CD19+CD24hiCD38hi Transitional B Cells and Autoantibody Production in SLE Patients. Front. Immunol..

[145] Deng Y., Zhao J., Sakurai D., Kaufman K.M., Edberg J.C., Kimberly R.P., Kamen D.L., Gilkeson G.S., Jacob C.O., Scofield R.H. (2013). MicroRNA-3148 Modulates Allelic Expression of Toll-Like Receptor 7 Variant Associated with Systemic Lupus Erythematosus. PLOS Genet..

[146] Shen N., Fu Q., Deng Y., Qian X., Zhao J., Kaufman K.M., Wu Y.L., Yu C.Y., Tang Y., Chen J.-Y. (2010). Sex-specific association of X-linked Toll-like receptor 7 (TLR7) with male systemic lupus erythematosus. Proc. Natl. Acad. Sci. USA.

[147] Wang C.-M., Chang S.-W., Wu Y.-J.J., Lin J.-C., Ho H.-H., Chou T.-C., Yang B., Wu J., Chen J.-Y. (2014). Genetic variations in Toll-like receptors (TLRs 3/7/8) are associated with systemic lupus erythematosus in a Taiwanese population. Sci. Rep..

[148] Brown G.J., Cañete P.F., Wang H., Medhavy A., Bones J., Roco J.A., He Y., Qin Y., Cappello J., Ellyard J.I. (2022). TLR7 gain-of-function genetic variation causes human lupus. Nature.

[149] Nickerson K.M., Wang Y., Bastacky S., Shlomchik M.J. (2017). Toll-like receptor 9 suppresses lupus disease in Fas-sufficient MRL Mice. PLoS ONE.

[150] Lartigue A., Courville P., Auquit I., François A., Arnoult C., Tron F., Gilbert D., Musette P. (2006). Role of TLR9 in Anti-Nucleosome and Anti-DNA Antibody Production in lpr Mutation-Induced Murine Lupus1. J. Immunol..

[151] Santiago-Raber M.L., Dunand-Sauthier I., Wu T., Li Q.Z., Uematsu S., Akira S., Reith W., Mohan C., Kotzin B.L., Izui S. (2010). Critical role of TLR7 in the acceleration of systemic lupus erythematosus in TLR9-deficient mice. J. Autoimmun..

[152] Stoehr A.D., Schoen C.T., Mertes M.M.M., Eiglmeier S., Holecska V., Lorenz A.K., Schommartz T., Schoen A.-L., Hess C., Winkler A. (2011). TLR9 in Peritoneal B-1b Cells Is Essential for Production of Protective Self-Reactive IgM To Control Th17 Cells and Severe Autoimmunity. J. Immunol..

[153] Yu P., Wellmann U., Kunder S., Quintanilla-Martinez L., Jennen L., Dear N., Amann K., Bauer S., Winkler T.H., Wagner H. (2006). Toll-like receptor 9-independent aggravation of glomerulonephritis in a novel model of SLE. Int. Immunol..

[154] Cosgrove H.A., Gingras S., Kim M., Bastacky S., Tilstra J.S., Shlomchik M.J. (2023). B cell-intrinsic TLR7 expression drives severe lupus in TLR9-deficient mice. JCI Insight.

[155] Celhar T., Yasuga H., Lee H.Y., Zharkova O., Tripathi S., Thornhill S.I., Lu H.K., Au B., Lim L.H.K., Thamboo T.P. (2018). Toll-Like Receptor 9 Deficiency Breaks Tolerance to RNA-Associated Antigens and Up-Regulates Toll-Like Receptor 7 Protein in Sle1 Mice. Arthritis Rheumatol..

[156] Fukui R., Saitoh S.-I., Kanno A., Onji M., Shibata T., Ito A., Onji M., Matsumoto M., Akira S., Yoshida N. (2011). Unc93B1 Restricts Systemic Lethal Inflammation by Orchestrating Toll-like Receptor 7 and 9 Trafficking. Immunity.

[157] Huh J.W., Shibata T., Hwang M., Kwon E.H., Jang M.S., Fukui R., Kanno A., Jung D.J., Jang M.H., Miyake K. (2014). UNC93B1 is essential for the plasma membrane localization and signaling of Toll-like receptor 5. Proc. Natl. Acad. Sci. USA.

[158] Kim Y.-M., Brinkmann M.M., Paquet M.-E., Ploegh H.L. (2008). UNC93B1 delivers nucleotide-sensing toll-like receptors to endolysosomes. Nature.

[159] Fukui R., Saitoh S., Matsumoto F., Kozuka-Hata H., Oyama M., Tabeta K., Beutler B., Miyake K. (2009). Unc93B1 biases Toll-like receptor responses to nucleic acid in dendritic cells toward DNA- but against RNA-sensing. J. Exp. Med..

[160] Leibler C., John S., Elsner R.A., Thomas K.B., Smita S., Joachim S., Levack R.C., Callahan D.J., Gordon R.A., Bastacky S. (2022). Genetic dissection of TLR9 reveals complex regulatory and cryptic proinflammatory roles in mouse lupus. Nat. Immunol..

[161] Hanna Kazazian N., Wang Y., Roussel-Queval A., Marcadet L., Chasson L., Laprie C., Desnues B., Charaix J., Irla M., Alexopoulou L. (2019). Lupus Autoimmunity and Metabolic Parameters Are Exacerbated Upon High Fat Diet-Induced Obesity Due to TLR7 Signaling. Front. Immunol..

[162] Fejtkova M., Sukova M., Hlozkova K., Skvarova Kramarzova K., Rackova M., Jakubec D., Bakardjieva M., Bloomfield M., Klocperk A., Parackova Z. (2022). TLR8/TLR7 dysregulation due to a novel TLR8 mutation causes severe autoimmune hemolytic anemia and autoinflammation in identical twins. Am. J. Hematol..

[163] Lind N.A., Rael V.E., Pestal K., Liu B., Barton G.M. (2022). Regulation of the nucleic acid-sensing Toll-like receptors. Nat. Rev. Immunol..

[164] Song H.-S., Park S., Huh J.-W., Lee Y.-R., Jung D.-J., Yang C., Kim S.H., Kim H.M., Kim Y.-M. (2022). N-glycosylation of UNC93B1 at a Specific Asparagine Residue Is Required for TLR9 Signaling. Front. Immunol..

[165] Majer O., Liu B., Woo B.J., Kreuk L.S.M., Van Dis E., Barton G.M. (2019). Release from UNC93B1 reinforces the compartmentalized activation of select TLRs. Nature.

[166] Ni H., Wang Y., Yao K., Wang L., Huang J., Xiao Y., Chen H., Liu B., Yang C.Y., Zhao J. (2024). Cyclical palmitoylation regulates TLR9 signalling and systemic autoimmunity in mice. Nat. Commun..

[167] Majer O., Liu B., Kreuk L.S.M., Krogan N., Barton G.M. (2019). UNC93B1 recruits syntenin-1 to dampen TLR7 signalling and prevent autoimmunity. Nature.

[168] Mishra H., Schlack-Leigers C., Lim E.L., Thieck O., Magg T., Raedler J., Wolf C., Klein C., Ewers H., Lee-Kirsch M.A. (2024). Disrupted degradative sorting of TLR7 is associated with human lupus. Sci. Immunol..

[169] Wolf C., Lim E.L., Mokhtari M., Kind B., Odainic A., Lara-Villacanas E., Koss S., Mages S., Menzel K., Engel K. (2024). UNC93B1 variants underlie TLR7-dependent autoimmunity. Sci. Immunol..

[170] Sato R., Shibata T., Tanaka Y., Kato C., Yamaguchi K., Furukawa Y., Shimizu E., Yamaguchi R., Imoto S., Miyano S. (2017). Requirement of glycosylation machinery in TLR responses revealed by CRISPR/Cas9 screening. Int. Immunol..

[171] Sun J., Duffy K.E., Ranjith-Kumar C.T., Xiong J., Lamb R.J., Santos J., Masarapu H., Cunningham M., Holzenburg A., Sarisky R.T. (2006). Structural and Functional Analyses of the Human Toll-like Receptor 3: ROLE OF GLYCOSYLATION*. J. Biol. Chem..

[172] Amith S.R., Jayanth P., Franchuk S., Siddiqui S., Seyrantepe V., Gee K., Basta S., Beyaert R., Pshezhetsky A.V., Szewczuk M.R. (2009). Dependence of pathogen molecule-induced Toll-like receptor activation and cell function on Neu1 sialidase. Glycoconj. J..

[173] Ramos-Martínez I., Ramos-Martínez E., Cerbón M., Pérez-Torres A., Pérez-Campos Mayoral L., Hernández-Huerta M.T., Martínez-Cruz M., Pérez-Santiago A.D., Sánchez-Medina M.A., García-Montalvo I.A. (2023). The Role of B Cell and T Cell Glycosylation in Systemic Lupus Erythematosus. Int. J. Mol. Sci..

[174] Cancro M.P. (2020). Age-Associated B Cells. Annu. Rev. Immunol..

[175] Mouat I.C., Goldberg E., Horwitz M.S. (2022). Age-associated B cells in autoimmune diseases. Cell. Mol. Life Sci..

[176] Jenks S.A., Cashman K.S., Zumaquero E., Marigorta U.M., Patel A.V., Wang X., Tomar D., Woodruff M.C., Simon Z., Bugrovsky R. (2018). Distinct Effector B Cells Induced by Unregulated Toll-like Receptor 7 Contribute to Pathogenic Responses in Systemic Lupus Erythematosus. Immunity.

[177] Wang S., Wang J., Kumar V., Karnell J.L., Naiman B., Gross P.S., Rahman S., Zerrouki K., Hanna R., Morehouse C. (2018). IL-21 drives expansion and plasma cell differentiation of autoreactive CD11chiT-bet+ B cells in SLE. Nat. Commun..

[178] Rubtsov A.V., Rubtsova K., Fischer A., Meehan R.T., Gillis J.Z., Kappler J.W., Marrack P. (2011). Toll-like receptor 7 (TLR7)–driven accumulation of a novel CD11c+ B-cell population is important for the development of autoimmunity. Blood.

[179] Aranburu A., Höök N., Gerasimcik N., Corleis B., Ren W., Camponeschi A., Carlsten H., Grimsholm O., Mårtensson I.-L. (2018). Age-associated B cells expanded in autoimmune mice are memory cells sharing H-CDR3-selected repertoires. Eur. J. Immunol..

[180] Dai D., Gu S., Han X., Ding H., Jiang Y., Zhang X., Yao C., Hong S., Zhang J., Shen Y. (2024). The transcription factor ZEB2 drives the formation of age-associated B cells. Science.

[181] Naradikian M.S., Myles A., Beiting D.P., Roberts K.J., Dawson L., Herati R.S., Bengsch B., Linderman S.L., Stelekati E., Spolski R. (2016). Cutting Edge: IL-4, IL-21, and IFN-γ Interact To Govern T-bet and CD11c Expression in TLR-Activated B Cells. J. Immunol..

[182] Nickerson K.M., Smita S., Hoehn K.B., Marinov A.D., Thomas K.B., Kos J.T., Yang Y., Bastacky S.I., Watson C.T., Kleinstein S.H. (2023). Age-associated B cells are heterogeneous and dynamic drivers of autoimmunity in mice. J. Exp. Med..

[183] Peng S.L., Szabo S.J., Glimcher L.H. (2002). T-bet regulates IgG class switching and pathogenic autoantibody production. Proc. Natl. Acad. Sci. USA.

[184] Woldemeskel B.A., Dykema A.G., Garliss C.C., Cherfils S., Smith K.N., Blankson J.N. (2022). CD4+ T cells from COVID-19 mRNA vaccine recipients recognize a conserved epitope present in diverse coronaviruses. J. Clin. Investig..

[185] Yasaka K., Yamazaki T., Sato H., Shirai T., Cho M., Ishida K., Ito K., Tanaka T., Ogasawara K., Harigae H. (2023). Phospholipase D4 as a signature of toll-like receptor 7 or 9 signaling is expressed on blastic T-bet + B cells in systemic lupus erythematosus. Arthritis Res. Ther..

[186] Kalliolias G.D., Basdra E.K., Papavassiliou A.G. (2024). Targeting TLR Signaling Cascades in Systemic Lupus Erythematosus and Rheumatoid Arthritis: An Update. Biomedicines.

[187] Patinote C., Karroum N.B., Moarbess G., Cirnat N., Kassab I., Bonnet P.A., Deleuze-Masquéfa C. (2020). Agonist and antagonist ligands of toll-like receptors 7 and 8: Ingenious tools for therapeutic purposes. Eur. J. Med. Chem..

[188] Cenac C., Ducatez M.F., Guéry J.-C. (2022). Hydroxychloroquine inhibits proteolytic processing of endogenous TLR7 protein in human primary plasmacytoid dendritic cells. Eur. J. Immunol..

[189] Ewald S.E., Lee B.L., Lau L., Wickliffe K.E., Shi G.-P., Chapman H.A., Barton G.M. (2008). The ectodomain of Toll-like receptor 9 is cleaved to generate a functional receptor. Nature.

[190] Schrezenmeier E., Dörner T. (2020). Mechanisms of action of hydroxychloroquine and chloroquine: Implications for rheumatology. Nat. Rev. Rheumatol..

[191] Guiducci C., Gong M., Xu Z., Gill M., Chaussabel D., Meeker T., Chan J.H., Wright T., Punaro M., Bolland S. (2010). TLR recognition of self nucleic acids hampers glucocorticoid activity in lupus. Nature.

[192] Deshmukh A., Pereira A., Geraci N., Tzvetkov E., Przetak M., Catalina M.D., Morand E.F., Bender A.T., Vaidyanathan B. (2024). Preclinical Evidence for the Glucocorticoid-Sparing Potential of a Dual Toll-Like Receptor 7/8 Inhibitor in Autoimmune Diseases. J. Pharmacol. Exp. Ther..

[193] Shisha T., Posch M.G., Lehmann J., Feifel R., Junt T., Hawtin S., Schuemann J., Avrameas A., Danekula R., Misiolek P. (2023). First-in-Human Study of the Safety, Pharmacokinetics, and Pharmacodynamics of MHV370, a Dual Inhibitor of Toll-Like Receptors 7 and 8, in Healthy Adults. Eur. J. Drug Metab. Pharmacokinet..

[194] Hawtin S., André C., Collignon-Zipfel G., Appenzeller S., Bannert B., Baumgartner L., Beck D., Betschart C., Boulay T., Brunner H.I. (2023). Preclinical characterization of the Toll-like receptor 7/8 antagonist MHV370 for lupus therapy. Cell Rep. Med..

[195] Tojo S., Zhang Z., Matsui H., Tahara M., Ikeguchi M., Kochi M., Kamada M., Shigematsu H., Tsutsumi A., Adachi N. (2020). Structural analysis reveals TLR7 dynamics underlying antagonism. Nat. Commun..

[196] Ishizaka S.T., Hawkins L., Chen Q., Tago F., Yagi T., Sakaniwa K., Zhang Z., Shimizu T., Shirato M. (2023). A novel Toll-like receptor 7/8–specific antagonist E6742 ameliorates clinically relevant disease parameters in murine models of lupus. Eur. J. Pharmacol..

[197] Murakami Y., Fukui R., Tanaka R., Motoi Y., Kanno A., Sato R., Yamaguchi K., Amano H., Furukawa Y., Suzuki H. (2021). Anti-TLR7 Antibody Protects Against Lupus Nephritis in NZBWF1 Mice by Targeting B Cells and Patrolling Monocytes. Front. Immunol..

[198] Achek A., Kwon H.K., Patra M.C., Shah M., Hong R., Lee W.H., Baek W.Y., Choi Y.S., Kim G.Y., Pham T.L.H. (2020). A peptide derived from the core beta-sheet region of TIRAP decoys TLR4 and reduces inflammatory and autoimmune symptoms in murine models. EBioMedicine.

[199] Moroni G., Frontini G., Ponticelli C. (2021). When and How Is It Possible to Stop Therapy in Patients with Lupus Nephritis: A Narrative Review. Clin. J. Am. Soc. Nephrol..

[200] Le Naour J., Kroemer G. (2023). Trial watch: Toll-like receptor ligands in cancer therapy. OncoImmunology.

[201] Srinivasa A., Tosounidou S., Gordon C. (2017). Increased Incidence of Gastrointestinal Side Effects in Patients Taking Hydroxychloroquine: A Brand-related Issue?. J. Rheumatol..

[202] Shang L., Wang L., Shi X., Wang N., Zhao L., Wang J., Liu C. (2020). HMGB1 was negatively regulated by HSF1 and mediated the TLR4/MyD88/NF-kappaB signal pathway in asthma. Life Sci..

[203] Xue J., Suarez J.S., Minaai M., Li S., Gaudino G., Pass H.I., Carbone M., Yang H. (2021). HMGB1 as a therapeutic target in disease. J. Cell Physiol..

